# Suppression of Scant Identifies Endos as a Substrate of Greatwall Kinase and a Negative Regulator of Protein Phosphatase 2A in Mitosis

**DOI:** 10.1371/journal.pgen.1002225

**Published:** 2011-08-11

**Authors:** Hélène Rangone, Eva Wegel, Melanie K. Gatt, Eirene Yeung, Alexander Flowers, Janusz Debski, Michal Dadlez, Veerle Janssens, Adelaide T. C. Carpenter, David M. Glover

**Affiliations:** 1 Department of Genetics, University of Cambridge, Cambridge, United Kingdom; 2 Laboratory of Mass Spectrometry, Institute of Biochemistry and Biophysics, Warsaw, Poland; 3 Department of Molecular Cell Biology, Faculty of Medicine, Katholieke Universiteit Leuven, Leuven, Belgium; National Institute of Diabetes and Digestive and Kidney Diseases, United States of America

## Abstract

Protein phosphatase 2A (PP2A) plays a major role in dephosphorylating the targets of the major mitotic kinase Cdk1 at mitotic exit, yet how it is regulated in mitotic progression is poorly understood. Here we show that mutations in either the catalytic or regulatory twins/B55 subunit of PP2A act as enhancers of *gwl^Scant^*, a gain-of-function allele of the Greatwall kinase gene that leads to embryonic lethality in *Drosophila* when the maternal dosage of the mitotic kinase Polo is reduced. We also show that heterozygous mutant *endos* alleles suppress heterozygous *gwl^Scant^*; many more embryos survive. Furthermore, heterozygous *PP2A* mutations make females heterozygous for the strong mutation *polo^11^* partially sterile, even in the absence of *gwl^Scant^*. Heterozygosity for an *endos* mutation suppresses this *PP2A*/*polo^11^* sterility. Homozygous mutation or knockdown of *endos* leads to phenotypes suggestive of defects in maintaining the mitotic state. In accord with the genetic interactions shown by the *gwl^Scant^* dominant mutant, the mitotic defects of Endos knockdown in cultured cells can be suppressed by knockdown of either the catalytic or the Twins/B55 regulatory subunits of PP2A but not by the other three regulatory B subunits of *Drosophila* PP2A. Greatwall phosphorylates Endos at a single site, Ser68, and this is essential for Endos function. Together these interactions suggest that Greatwall and Endos act to promote the inactivation of PP2A-Twins/B55 in *Drosophila*. We discuss the involvement of Polo kinase in such a regulatory loop.

## Author Summary

Progression through mitosis requires the addition of phosphate groups onto specific proteins by enzymes collectively known as mitotic protein kinases. At the end of mitosis, these phosphates are removed by protein phosphatases. Whereas we know quite a lot about the mitotic protein kinases, we know much less about the phosphatases. Here we used the fruit fly *Drosophila* as a model organism to identify a pathway regulating a phosphatase required for mitotic exit. Using mutations in genes for this pathway in the fly and by depleting levels of corresponding proteins from cultured cells, we established the relationships between the gene products. This has revealed that Greatwall mitotic kinase works in concert with the protein Endos to antagonise Protein Phosphatase 2A (PP2A). Specifically, Greatwall and Endos affect the activity of a particular form of PP2A that is associated with only one of the four different regulatory subunits found in *Drosophila*. We found that phosphorylation of Endos at a defined position by Greatwall kinase is required for its function. Together this provides genetic evidence that the Greatwall mitotic kinase inhibits the PP2A phosphatase required for mitotic exit thus complementing biochemical experiments using frog eggs and indicating the universality of this mechanism.

## Introduction

Greatwall (Gwl) is a highly conserved protein kinase that has been shown to have important mitotic functions in *Drosophila*, *Xenopus* and humans [1]–[10]. Loss-of-function alleles of the *Drosophila* gene *Greatwall (gwl)* lead to cell cycle delay at the G2-to-M transition and mitotic chromosomes show unusual states of condensation [1]. Depletion of the Gwl kinase from cultured *Drosophila* cells resulted in similar mitotic delays and a characteristic phenotype of conjoined chromatids scattered upon mitotic spindles that were elongated as if in anaphase B [2]. *Xenopus* Gwl is activated by MPF and is required for M-phase entry. Removal of Gwl from CSF *Xenopus* extracts leads to an unusual mitotic exit in which cyclins remain undegraded but Cyclin-dependent kinase 1 (Cdk1) is inactivated by phosphorylation at Thr14 and Tyr15 [3], [5]. Thus it seemed that Gwl could facilitate activation of Cdk1 *via* the phosphorylation-dependent activation of Cdc25 and inhibition of Myt1/Wee1 [5]. That Gwl might be regulating a protein phosphatase was suggested by the finding that addition of the phosphatase inhibitor okadaic acid re-enables Gwl-depleted interphase extracts to enter M phase [5]. This phosphatase proved to be PP2A, a heterotrimeric protein comprising a catalytic C subunit, a structural A subunit, and one of several regulatory B subunits, in this case the B55 regulatory subunit. Inhibition or depletion of PP2A from mitotic extracts rescued the inability of Gwl-depleted extracts to enter M phase [6], [7]. PP2A-B55 has been shown to be a major protein phosphatase responsible for reversing Cdk1-mediated phosphorylation in both *Drosophila* [11] and *Xenopus* [12]. Two recent biochemical studies have identified two related substrates of Greatwall kinase, α-Endosulfine (Ensa) and Arpp19, as inhibitors of PP2A in *Xenopus* egg extracts [13], [14].

The first *gwl* allele to be identified in *Drosophila* was a gain-of-function allele given the name *Scant* [15]. When heterozygous with one mutant copy of *polo* (and one copy of *polo^+^*), the gene for the mitotic Polo kinase, *Scant* causes females to produce embryos that have greatly reduced viability. Because the *Scant* mutation causes no recessive phenotype, it could not be mapped precisely until recessive alleles of its gene were identified. This was achieved by inducing revertants of the *polo-Scant* sterility; three of these are simple recessive mutations in *gwl* [4]. Two of these *gwl* alleles showed mutant phenotypes in larval neuroblasts similar to those previously described [1] and one allele, a female-specific germline splicing mutant, showed only female sterility. The oocytes of females hemizygous for this allele, *gwl^Sr18^*, fail to arrest in metaphase of the first meiotic division and both homologues and sister chromatids separate on elongated meiotic spindles with little or no segregation. The *Scant* mutation results from a single amino acid change, K97M; this shows dramatically increased activity of the kinase towards artificial substrates. Surprisingly, the defect in embryos from *polo Scant/+ +* mothers is simple and single; centrosomes tend to detach from the nuclear envelope during migration during the syncytial divisions [4], leading to aberrations in subsequent mitoses. Since *Scant* has no phenotype when there are two wild-type copies of *polo*, and *polo* itself is recessive, this observation implies that the level of functional Polo (or one of its targets) is reduced in the presence of *Scant*; and that in turn suggests that maintaining centrosome-nuclear envelope conjunction is the Polo function requiring the highest level of Polo activity, since everything else that Polo is known to promote still occurs normally in these embryos. The highly sensitized *polo Scant/+ +* genotype therefore allows us to probe this specific role of Polo protein.

*Scant* revertants also included two *polo^+^* duplications, consistent with the requirement for reduced *polo* function in order to see reduced fertility in the presence of *gwl^Scant^*, and a third suppressor genotype that is a large deficiency elsewhere. We now show that this latter suppression is due to reduced dosage of *endos*, which encodes the *Drosophila* α-Endosulfine, previously shown to be required for oocytes to progress to metaphase of the first meiotic division and to regulate levels of Twine, the germline-specific Cdc25 phosphatase, and Polo kinase in those oocytes [16]. We also show that mutations in the catalytic and B55 (*twins* in *Drosophila*) regulatory subunits of PP2A enhance the *polo Scant* maternal effect suggesting antagonistic effects of Endos and PP2A. We find that down-regulation of *endos* in tissue-culture cells leads to abnormal mitotic cells having elevated cyclin B and elongated spindles on which chromosomes are highly scattered and have not undergone sister chromatid separation. This phenotype is suppressed by simultaneous depletion of the catalytic or structural subunits of PP2A and by its *twins* regulatory B subunit but not by depletion of its other regulatory B subunits. These genetic interactions in *Drosophila* are in accord with the recently published biochemical studies in *Xenopus* [13], [14]. Indeed we find that *Drosophila* Greatwall kinase phosphorylates Endos at a single site and mutation of this residue perturbs Endos's mitotic function. Thus the major aspects of the Greatwall – Endos – PP2A regulatory circuit appear to be conserved in evolutionarily diverged metazoans.

## Results

### *gwl^Scant^* is suppressed by *endos* and enhanced by the PP2A mutants *twins* and *mts*

**Figure 1:**
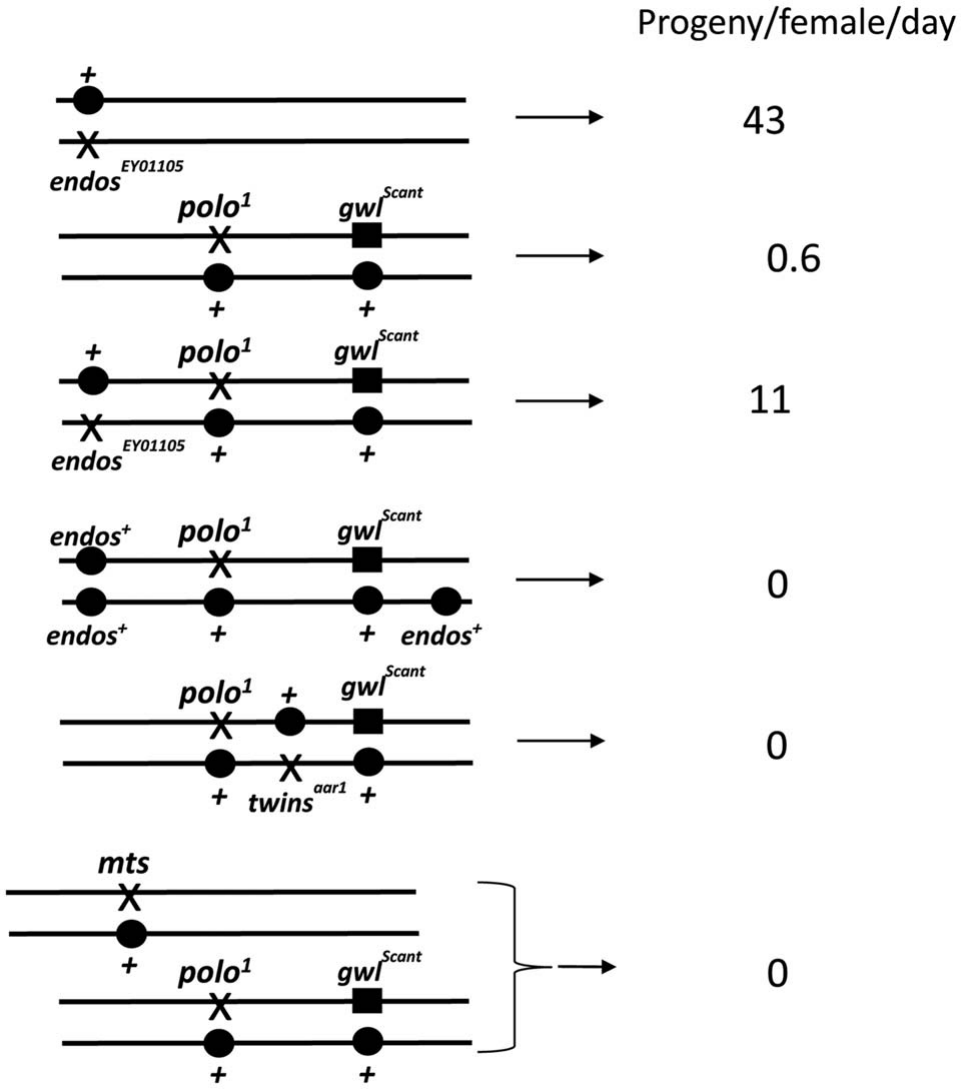
Genotypes that suppress or enhance *polo^1^ gwl^Scant^* fertility. *polo^1^ gwl^Scant^*/+ + females produce embryos with very poor survival because mitotic defects follow the primary defect of centrosome detachment during migration around the nuclear envelope in interphase. Mutations in *endos* suppress this lethality. An additional transgenic copy of *endos^+^* enhances this lethality as do mutations in *twins* (PP2A B55 subunit) and *mts* (PP2A catalytic subunit). Survival is expressed as the number of adult progeny produced per day per mother of the indicated genotype cultured on enriched fly food. Data from all control genotypes are presented in Table 1.

To gain insight into Greatwall function, we first examined the cytology of the above third site suppressor in larval salivary gland chromosomes and found it to be a deficiency on 3L: *Df(3L)Sr5*, *70C7-15;70F3-7*. Identification of the suppressor was facilitated by recombining the *gwl^Scant^* mutation and *polo^1^* or *polo^11^* onto the same chromosomes [4]. Tests with independent large deficiencies heterozygous with this *polo^1^ gwl^Scant^* chromosome confirmed that the original suppression is due to haplo-insufficiency of a gene(s) in the 70C7-70D5 interval. Tiling this interval with small deficiencies mapped the major suppressor to the 70C7-15 interval within which lies the *endos* gene previously shown to encode a small phospho-protein, α-endosulfine or Endos. In a direct test of the ability of *endos* mutants to act as dominant suppressors, we found that *endos/polo^1^ gwl^Scant^* females (one copy of *endos^+^*; Figure 1, Table 1) are reasonably fertile (11 adult progeny per female per day for *endos^EY01105^*), whereas *+ +/polo^1^ gwl^Scant^* females (two copies of *endos^+^*) are nearly sterile (0.6 adult progeny per female per day). We then asked what the consequence of increasing the gene dosage of *endos^+^* is by testing the fertility of *polo^1^ gwl^Scant^/+ +* females also heterozygous for an *endos^+^* transgene (see below); three copies of the wild-type *endos* gene enhanced the *polo^1^ gwl^Scant^* phenotype (Figure 1, Table 1), *i. e.*, there were no adult progeny at all, or, indeed, any egg development.

**Table 1:** Interactions affecting female fertility examined in this study expressed as progeny per female per day for *mutation/indicated genotype*.

Mutation	/*polo^1^*	*/polo^1^ Scant*	rescue ratio	/*polo^11^*	*/Scant*	*/polo^11^ Scant*
*Oregon R (wt)*		0.6				0
*endos^EY01103^*	41	9	15x	46	43	0.9
*endos^EY01105^*	43	11	18x	53	45	2.3
*GFP-polo^+^/X*	28	27				14
*endos^+^ + endos^+^ transgene*		0				0
*tws^aar1^*	34	0	0	1.2	19	0
*tws^P^*	42	0	0	0.5	6	0
*mts^E2202^*	28	0	0	8	0.04	0
*endos^EY01103^ tws^aar1^*	10	0		9		
*endos^EY01103^ tws^P^*	38	0		0.3		
*endos^EY01105^ tws^aar1^*	34	0.2		24		
*endos^EY01105^ tws^P^*	30	0		4		
*mts^E2202^ endos^EY01103^*	29	0.02		4		
*mts^E2202^ endos^EY01105^*	12	0		5		

Tests were performed to maximise recovery of all adult progeny from the first 15 days of egg laying (see Materials and Methods). Each value is the average from three separate females whose progeny developed on rich medium; an additional three separate progenies that developed on less rich food gave equivalent results^a^.

a This particular set of data was chosen because all of the fertility tests were done within the same time frame; *endos/polo^1^ Scant* females can give 20–30 progeny/female/day but they typically lose fertility sooner with age than controls do.

Several laboratories have shown that the *Xenopus* counterpart of Greatwall kinase acts by down-regulating PP2A activity (see Introduction; [5]–[7]). These findings therefore led us to confirm our crude observations that PP2A mutants enhance the infertility of *polo^1^ gwl^Scant^*. We found that each of two independent mutations in *twins* that encodes the B55 regulatory subunit, *tws^aar1^* [17] and *tws^P^* [18], were completely female sterile when heterozygous with *polo^1^ gwl^Scant^* (Figure 1, Table 1). Furthermore, mutation in the catalytic C subunit, *microtubule star* (*mts^E2202^*; [19]), also led to complete sterility when heterozygous in *polo^1^ gwl^Scant^*/+ + females. Thus, in contrast to *endos*, mutations in the PP2A subunits enhance the Scant phenotype. In addition, *endos/+* also slightly suppresses the sterility of *PP2A/polo gwl^Scant^* females in an allele-specific manner: *endos^EY01105^ tws^aar1^/polo^1^ gwl^Scant^* (stronger *endos* allele, weaker *tws* allele, see Materials and Methods) females produced a few progeny (Table 1) whereas *endos^EY01103^ tws^P^/polo^1^ gwl^Scant^* (weaker *endos* allele, stronger *tws* allele) produced eggs that died without any significant embryonic development. Together these genetic interactions suggest that *endos* and *tws* (PP2A-B55) have opposing roles in regulating the dominant effect of *gwl^Scant^* when Polo function is reduced.

### *endos* and *twins* have opposing roles in regulating a critical function of *polo*

For suppression, here of the *polo gwl^Scant^* sterility by *endos*, the prediction is that there will be no effect when the two components are tested separately, and indeed both the *endos* alleles are fully fertile trans-heterozygous either with *polo^1^* or *polo^11^* alone or with *gwl^Scant^* alone (Table 1). In these genotypes a single mutant copy of either *endos^EY01103^* or *^EY01105^* has no effect upon Polo levels in contrast to the reduction in Polo levels seen in oocytes/ovaries from homozygous *endos* females ([16], Figure S1A). Enhancement, on the other hand, here of the *polo gwl^Scant^* sterility by *PP2A*, offers the possibility of asking which component is the more important. Although no reduction in fertility was observed in *polo^1^*/*PP2A* (*tws* or *mts*) transheterozygotes, these *PP2A* mutations have reduced fertility when transheterozygous with the amorphic allele *polo^11^* or with the dominant *gwl^Scant^*. Moreover, the stronger *tws^P^* allele (see Materials and Methods) reduces fertility of heterozygous *polo^11^* more than the weaker *tws^aar1^* allele.

As expected, reducing the dosage of *endos^+^* has little effect on the already-fertile combinations of *PP2A* (*tws* or *mts*) with *polo^1^*, but such reduction generally improves the fertility of *PP2A/polo^11^* females; indeed, the *endos^EY01105^* (stronger allele) *tws^aar1^* (weaker allele)/*polo^11^* combination has nearly normal fertility. Thus, even in the absence of the confounding *gwl^Scant^* mutation, Endos and PP2A-B^Twins^ can still be seen to have opposing roles.

Together, these genetic interactions suggest that *endos* and *tws* (PP2A-B55) have opposing roles in responding to the dominant effect of *gwl^Scant^*. Since Gwl has been shown to down-regulate PP2A activity in *Xenopus*, this suggests that Gwl and Endos might also function together towards this end in *Drosophila*.

### Mutations in *endos* lead to an elevated mitotic index with anaphase bridges in *Drosophila* larval neuroblasts

**Figure 2:**
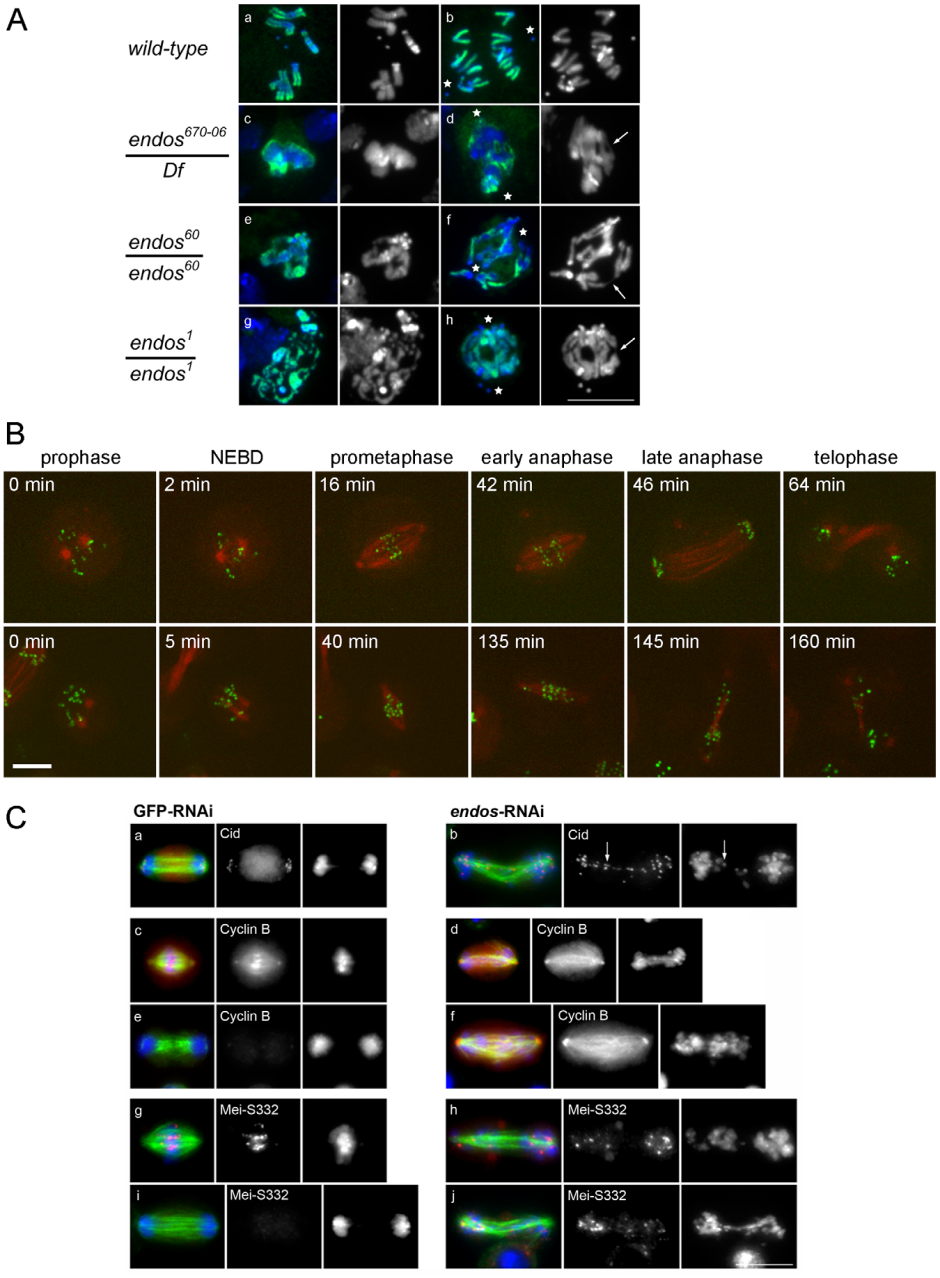
Loss of *endos* function leads to defective mitotic progression. A. Mitotic figures in both wild type and in the indicated *endos* mutant larval brains. Metaphase (left) and anaphase (right) neuroblasts are shown. Because mitotic chromosomes in the *endos* mutants are under-condensed (panels c, e, and g), it was necessary to counter-stain for phospho-Histone H3 to identify mitotic cells. Abnormal mitotic chromosome condensation is extreme in some cells (*e.g.* panel g) where the mitotic stage of the chromatin mass is difficult to classify. In the rest, nuclei positive for phospho-Histone H3 staining and showing a single chromosome mass were classified as metaphase; those with phospho-Histone H3 staining where the bulk of chromatin was distributed between two masses or in which distinct chromosomes could be seen with both polar and bridging configurations were classified as anaphase. The most striking phenotype is of chromatin bridges during anaphase (panels d, f and h); these are unambiguously bridges when the chromatin has been stretched. Stars mark poles and arrows mark stretched bridges – there are many more probable bridges in these figures than the few marked with arrows. Scale bar represents 10 µm. Details of the *endos* alleles analysed here and the levels of Endos protein they express are shown in Figure S1. Quantitation of the increased ratio of metaphase∶anaphase figures and of anaphase defects for these mutant combinations based on the above criteria are given in Table S1. (B) Time lapse imaging of DMEL cells expressing GFP-Cid and β-tubulin-mRFP. The control cell is going through mitosis from prophase to telophase with a prometaphase duration of approximately 40 minutes (prometaphases in control cells do not exceed 58 minutes). The cell depleted for Endos is going through a prolonged prometaphase of approximately 130 minutes (prometaphases in all cells depleted for Endos exceed 50 minutes) and exhibits dispersed Cid signals on its spindle. Timings of this prolonged prometaphase-like state for all cells analysed by time lapse imaging are given in Table S3. (C) Localisation of Cid, Cyclin B and MeiS332 after GFP and *endos* RNAi in cell culture. Analysis of fixed preparations indicates that Endos depletion induces abnormally long spindles upon which chromosomes fail to congress (b, f, h, and j). Cells were stained to reveal the centromeric proteins Cid (panel a and b) and MeiS332 (panel g, h, i and j) and the regulatory protein Cyclin B (panel c, d, e and f) together with tubulin and DNA DAPI staining. Paired centromeres (arrows), high levels of Cyclin B and the presence of the Shugoshin protein MeiS332 are found in cells depleted for Endos. Scale bar represents 10 µm. Quantitation of mitotic index and proportions of cells showing major defects in spindle morphology and chromosome scattering are given in Table S2.

The above findings led us to ask whether loss of *endos* function has the same consequences for mitotic progression that loss of *gwl* does. Because only maternal-effect *endos* phenotypes had been reported for female meiosis and in the rapid mitotic cycles of syncytial embryos [16], we examined the zygotic mitotic phenotypes of *endos* mutants to determine whether they are similar to *gwl*. Flies hemizygous for *endos^67006^*, a P-element insertion in the 5′ region of the *endos* gene [20], had multiple defects typical of abnormalites in cell cycle progression: shrivelled wings, missing thoracic bristles, irregular abdominal bristles, disrupted tergites and legs, and male and female sterility. These mutant phenotypes were all reverted following precise excision of the P element responsible for the mutation, a procedure that also generated additional alleles (*endos^79^*, *endos^60^* and *endos^1^*) from imprecise excisions (Figure S1B). A transgenic rescue construct containing the *endos* gene, but not one carrying the divergent transcription unit (*CG6650*), rescued the mutant phenotypes and sterility of all alleles tested. The new *endos* alleles generated in this way were similar to the original P-element mutant with respect to their external phenotype and sterility, with the exception of *endos^79^*; here, males are fertile, suggesting that it is a weaker hypomorph. Western blotting, using an antibody raised against the full-length Endos protein, showed that there was no detectable protein present in larval neuroblasts isolated from the *endos^1^* mutant (Figure S1C) in accord with its partial deletion of the ORF (Figure S1B). Protein levels in *endos^60^* were similar to the parental *endos^67006^* whereas higher levels were found in *endos^79^*. Larval brains from homozygous *endos^1^* null larvae were approximately half the wild-type size, suggesting cell cycle defects [21]. Squashed preparations of larval neuroblasts revealed an almost doubling of the mitotic index and an increase in the proportion of metaphase∶anaphase figures (4∶1 in wild-type compared to 11∶1 in hemizygous *endos^1^*; Table S1). The few anaphases present showed a high incidence of anaphase bridging; this was never seen in wild-type cells and was proportional to the strength of the mutant allele examined (Figure 2A). Some mitotic cells were very difficult to score, because they did not appear to be fully in mitosis judged by the low level of condensation of their chromosomes; nevertheless, they were positive for phospho-histone H3, an indicator of mitotic activity of the Aurora B kinase (Figure 2A). Taken together these phenotypes are very similar to those of recessive alleles of *gwl* [1], [4]. This is in accord with the two proteins acting as positive mediators of the same pathway and the ability of reduced dosage of *endos* to suppress the dominant phenotype of *polo^1^ gwl^Scant^*.

### Mitotic defects of *endos* RNAi in *Drosophila* cell culture

Defective mitoses accrue in mitotic mutants of *Drosophila* as the maternally provided wild-type proteins are gradually depleted during development. The kinetics of this depletion depends upon the particular protein and also upon the nature of the mutant allele. It is therefore not uncommon for mutant phenotypes to be complicated by secondary defects that follow from the primary one. Therefore, as an alternative way to examine the mitotic effect of *endos*, we examined cultured DMEL cells following depletion of Endos by RNAi. A 72 h treatment of cells with dsRNA targeting the *endos* gene eliminated >90% of the Endos protein and led to a decreased proportion of cells in G1 (from 53.0% in GFP depleted control cells to 38.7% in Endos depleted cells; Figure S2). Immunostaining of fixed preparations also revealed an increase in the mitotic index. This was associated with a substantial reduction of the proportion of mitotic cells in telophase and cytokinesis (39% in control cells *vs.* 15% in *endos* RNAi cells; Table S2). Time lapse imaging of cells expressing GFP-Cid (*Drosophila* CENP-A; [22]) and β-tubulin-mRFP revealed that, following depletion of Endos, prometaphase was greatly prolonged in comparison with control cells (Figure 2B, Table S3). The centromeres of chromosomes did not congress fully and became scattered as the spindle eventually began to elongate in what appeared to be an attempted anaphase. This led in some cases to unequally distributed chromosomes (Figure 2B, Video S1 and Video S2). This scatter was also observed in immunostained fixed preparations in which a high proportion (84%) of mitotic cells following *endos* RNAi treatment cells showed mis-aligned and scattered chromosomes frequently on elongated spindles (Figure 2C, Table S2). Whereas in control cells depleted with dsRNA targeting the GFP gene (GFP-RNAi), the *Drosophila* Shugoshin, MeiS332 (reviewed in [23]), is lost from centromeres at the metaphase-anaphase transition, in *endos* RNAi-treated cells it was still present at the centromeric regions of many of the scattered chromosomes (Figure 2C). Furthermore, whereas control cells with elongated spindles had undergone Cyclin B destruction at the metaphase-anaphase transition, *endos* RNAi-treated cells with elongated spindles and scattered chromosomes still had high levels of Cyclin B (Figure 2C). The presence of at least some conjoined centromeres and presence of Cyclin B suggests a prolonged checkpoint response that markedly delays APC/C-dependent processes at the metaphase – anaphase transition. Such phenotypes are extremely similar to the phenotype we reported for the depletion of Greatwall kinase in cultured cells ([2], Figure S4 in [4]). Thus this assay is also in accord with Greatwall and Endos participating in the same pathway. Further support for this conclusion comes from the similar patterns of sub-cellular localisation of Endos (Figure S3A, Figure S3B) and Greatwall (Figure S7 in [4]).

### *endos* knockdown is suppressed by *twins* knockdown

The *endos* mutations used in the genetic studies are homozygous viable but have delayed eclosion relative to their balancer siblings. We found that this eclosion delay is rescued in *endos +/endos tws* flies. However, the rescued females are still completely sterile, so for phenes other than developmental timing the suppression is weak. Moreover, there was little effect of these *endos* mutations, even as homozygotes, upon the lethal phase of *tws* mutants. Notwithstanding the multiple developmental roles of PP2A and the pleiotropy of its phenotypes, there are also considerable difficulties in making comparisons of mutant mitotic phenotypes in pupae because of the potential for differential perdurance of maternal protein during the first week of development. Thus we turned to cell culture to examine the consequences of co-depleting either Greatwall or various PP2A subunits upon phenotypes characteristic of *endos* depletion.

**Figure 3:**
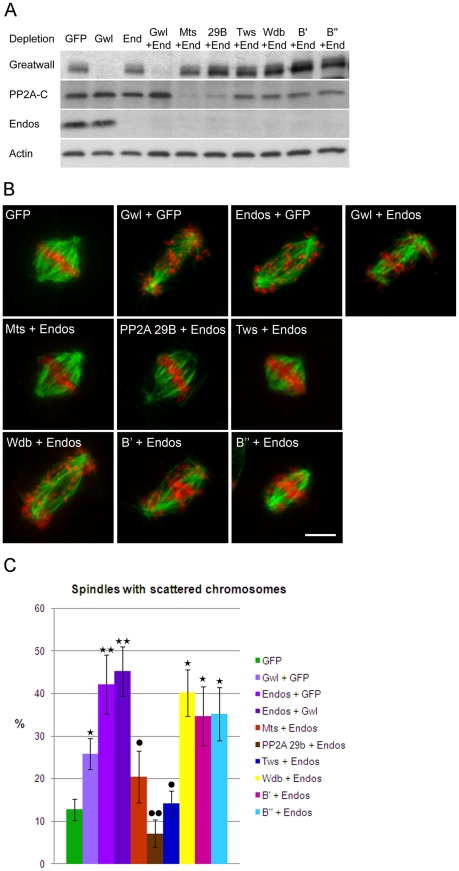
Mitotic defects of *endos* knockdown are suppressed by *PP2A*-*twins/B55* knockdown. (A) Levels of depletion of Greatwall, Microtubule-star and Endos in DMEL cells. Levels of proteins were visualised by Western blot after single or double depletion of DMEL cells for GFP (control depletion), Greatwall (Gwl), Endos (End) and the various subunits of PP2A: Microtubule Star (*Mts*), 29B, *twins* (Tws), B′ and B″. Extracts were analysed with antibodies detecting Greatwall, the catalytic subunit of PP2A (PP2A-C), Endos and Actin (loading control). Note that unfortunately we do not have antibodies against the A structural or any of the B regulatory subunits. Their mRNAs are, however, effectively depleted under these conditions [33]. Note also that knockdown of the A subunit destabilises the C subunit as previously reported [33]. (B) Representative mitotic figures after depletion of Greatwall, Endos and the various subunits of PP2A. DMEL cells were stained for α-tubulin in green and DAPI in red. Prometaphases with dispersed chromosomes are the major mitotic defects observed after depletion of either Greatwall or Endos. This phenotype is suppressed by co-depletion of Endos and the PP2A-*twins* subunit. Scale bar represents 5 µm. (C) The above mitotic defects following RNAi treatment were scored as proportions of prometaphase figures with dispersed chromosomes. The *endos* depletion phenotype is suppressed by knocking down the PP2A *twins* B subunit but not the B subunit encoded by *widerborst*, *B′ or B″*. It is also suppressed by knockdown of the catalytic C subunit (Mts) and the structural A subunit (*PP2A 29B*). Error bars represent sem of three independent experiments. P values are from a Student's-T test with * or • = 0.05<p<0.01; ** or •• = 0.01<p<0.001 (non-significant differences are not shown). * indicates comparison with GFP control and • indicates comparison with Endos depletion. A minimum of 600 prometaphases were scored per treatment.

We chose to quantitate the dispersion of chromosomes on prometaphase figures because this phenotype is the most characteristic of the *endos* knockdown (Figure 3). *gwl* knockdown gives a similar, but less dramatic, phenotype. Upon the double knockdown of *endos* and *gwl* there is only a slight increase in the severity of the phenotype (Figure 3C) suggesting that Endos and Greatwall are acting in the same pathway to control chromosome dispersion on the spindle. In contrast, the *endos* depletion phenotype was suppressed by co-depletion of the B subunit of PP2A (B55) encoded by *twins* but not by depletion of any of *Drosophila's* other three B subunits, Widerborst, B′ or B″. Depletion of either the catalytic (*mts*) or structural (*PP2A 29B*) subunits also suppressed the *endos* knockdown phenotype (Figure 3C). Cells depleted of either *mts* or *PP2A 29B* in addition to *endos* were not completely normal in distribution of microtubules; they had many long microtubules that were not captured by the spindle (Figure S4B). Nevertheless, the rescue of spindle morphology in doubly-depleted cells was striking. Another phenotype of Endos depletion, telophase cells with chromosome bridges, was also suppressed by simultaneously depleting the Twins B subunit of PP2A (Figure S4C, Figure S4D). That Endos knockdown is suppressed by knockdown of components of the PP2A-Twins heterotrimer is in accord with the genetic interactions of the gain-of-function *gwl^Scant^* allele with *mts* and *tws* mutants suggesting that Endos functions by opposing PP2A function.

Because the level of Polo has been reported to be greatly diminished in *Drosophila* oocytes from *endos* homozygous mutant females [16], we assayed whether Polo levels are affected after depletion of Endos in cell culture. In the conditions used for our assay neither single depletion of Endos or Greatwall or combined depletions of Endos and PP2A subunits lead to any major effect on Polo levels (Figure S4A).

### Endos is a Greatwall kinase substrate

**Figure 4:**
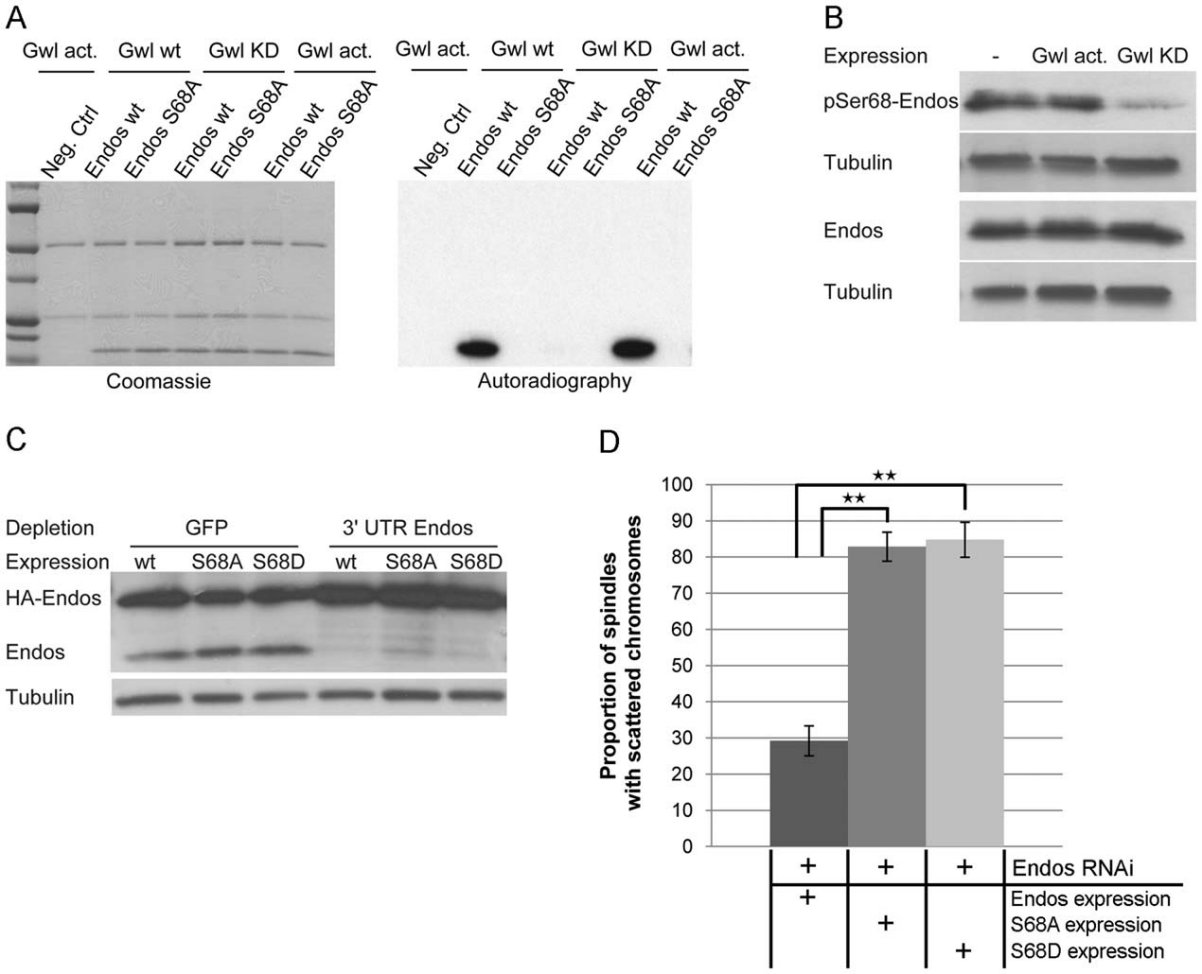
Greatwall phosphorylates Endos at Ser68. (A) Endos is phosphorylated at Ser68 by Greatwall. *In vitro* phosphorylation assays were performed using Myc-Greatwall wild type (Gwl wt), Myc-Greatwall kinase dead (Gwl KD, K87R) or Myc-Greatwall *Scant* hyperactive form (Gwl act., K97M) immunoprecipitated from cell extracts with Endos as substrate. Endos wild type (Endos wt) and mutated for S68A (Endos S68A) were expressed in bacteria as GST tagged proteins, purified on GS beads and cleaved from GST with thrombin protease. The reactions were resolved by SDS-PAGE; the gel was stained with Coomassie Blue (left panels) and the ^32^P-labeled proteins were visualized by autoradiography (right panels). The Greatwall wild type and hyperactive forms strongly phosphorylate Endos. This phosphorylation activity is drastically reduced in the presence of the kinase-dead form of Greatwall. Phosphorylation of Endos is abolished when Ser68 is mutated into Ala. (B) Greatwall phosphorylates Endos at Ser68 in cultured cells. Cells stably expressing Myc-Greatwall kinase dead (Gwl KD, K87R) or Myc-Greatwall hyperactive form (Gwl act., K97M) or non-expressing cells were treated with okadaic acid at 25 nM for 2 hours before preparation of extracts. Extracts were analysed on independent Western blots using antibodies detecting either Endos phosphorylated at Ser68 (pSer68-Endos, upper panels) or Endos (lower panels) and tubulin (loading control). A reduced level of Endos phosphorylation is observed in cells expressing the kinase dead form of Greatwall. (C) Levels of depletion of endogenous Endos and of expression of Endos Ser68 mutants in DMEL cells. Levels of proteins are visualised by Western blot after depletion of cells for GFP (control depletion) or endogenous Endos using dsRNA against the 3′UTR of the *endos* gene. Following depletion, several constructs of HA-tagged Endos were expressed by transient transfection: HA-Endos wild-type (wt), HA-Endos S68A (S68A), HA-Endos S68D (S68D). Extracts were analysed with antibodies detecting Endos (detecting both endogenous Endos (15 kDa) and HA-tagged Endos (24 kDa)) and Tubulin (loading control). (D) Incidence of the *endos* phenotype in prometaphases after depletion of Endos and overexpression of Endos or Endos Ser68 mutants. The phenotype in cells expressing exogenous Endos or its mutants is normalised relative to depleted cells on the same coverslip that are not expressing exogenous protein (the proportion of defects in cells not expressing exogenous protein is set to 100%). Error bars represent sem of three independent experiments. P values are from a Student's-T test with ** = 0.05<p<0.01 (non-significant differences are not shown); a minimum of 600 prometaphases were scored per treatment. Exogenous Endos expression rescues the prometaphase phenotype caused by Endos depletion whereas the S68 mutants do not rescue.

The above findings led us to ask whether Greatwall and Endos might be working together because Endos is a substrate of Greatwall. To this end we immunoprecipitated Greatwall protein from extracts of *Drosophila* cell lines stably expressing Myc-tagged wild-type Greatwall (Gwl wt), Scant mutant Greatwall (Gwl act: K97M), or kinase dead Greatwall (Gwl KD: K87R) to determine whether these enzymes were able to phosphorylate Endos expressed as a GST-fusion protein in bacterial cells and released by thrombin protease after affinity purification (Figure 4A, Figure S5A). The wild-type and activated (K97M) forms of Greatwall were able to phosphorylate wild-type Endos but not a variant of Endos in which Serine 68 was mutated to an Alanine residue; this single mutation totally abolished Greatwall kinase-mediated phosphorylation of Endos (Figure 4A). Mass spectrometric analysis also identified Serine 68 as the single site phosphorylated *in vitro* by Greatwall in the Endos protein. It lies in a region strongly conserved with the analogous Greatwall kinase phosphorylation site in *Xenopus* Ensa [13], [24].

Several lines of evidence suggest that Endos is phosphorylated at Serine 68 *in vivo*. Firstly, we were able to detect this modification in cell extracts using an antibody directed specifically against an Endos peptide phosphorylated at Serine 68 ([13], Figure 4B). Endos phosphorylated at Serine 68 (P-Ser68 Endos) could be weakly detected in extracts of asynchronous cells and more strongly after okadaic acid treatment (Figure S5B, Figure S5C). This treatment also resulted in an increase in total Endos levels suggesting that the phospho-form might show increased stability (Figure S5B, Figure S5C). Expression of the hyperactive Greatwall mutant (Gwl act: K97M) gave no elevation of P-Ser68 Endos above that seen following okadaic acid treatment (Figure 4B). Expression of a kinase-dead mutant form of Greatwall, however, resulted in a reduced level of P-Ser68 Endos, suggesting that it competes with endogenous Greatwall in phosphorylating this site (Figure 4B). Treatment of cells with varying concentrations of okadaic acid followed by electrophoresis of the cell extracts on conventional SDS-containing gels or SDS gels also containing Phos-tag (Figure S5B, Figure S5C) revealed that Endos is subject to at least three different phospho-modifications. The sensitivity of one of these sites to low concentrations of okadaic acid suggests that it is normally dephosphorylated by PP2A but P-Ser68 and at least one other phosphorylation are sensitive to two other protein phosphatases. Together this suggests that Endos is phosphorylated *in vivo* at Serine 68 and at least two other amino acid residues. The kinases responsible for these phosphorylations are quite likely to be PKA and CDK or kinases related to them since these enzymes are known to phosphorylate Endos in *Xenopus* [13].

To determine whether Serine 68 is essential for the function of Endos in cultured cells, we mutated this residue to Alanine (S68A). We used dsRNA directed against the 3′ non-coding sequence of endogenous *endos* to deplete its protein from cultured cells leading to the characteristic *endos* depletion phenotype described above (Figure 4C, Figure 4D). Complete rescue of the phenotype was achieved by transfecting these cells with a construct expressing HA-tagged wild-type Endos protein from the Actin 5C promoter. In contrast, the S68A Endos construct was completely unable to rescue the phenotype. We then asked whether substitution of an acidic amino acid for Serine 68 mimics the effects of phosphorylation but substitution by an Aspartic acid residue (S68D) also failed to rescue depletion of the wild-type protein (Figure 4C, Figure 4D). The inability of the S68D mutant to rescue the phenotype might reflect either the degree to which the acidic amino acid can mimic a phosphate residue at this site or the possibility that the ability to cycle between phosphorylated and dephosphorylated states is required at different mitotic stages (see also below). Nevertheless, together these experiments strongly suggest that Greatwall-mediated phosphorylation of Endos on Serine 68 is required for its function.

## Discussion

We identify *endos* mutations as heterozygous suppressors of the dominant mutant phenotype of *polo^1^ gwl^Scant^*. This suggests that Greatwall and Endos promote the same mitotic pathway. In accord with this we find that the consequences of loss of *gwl* and of *endos* function in mitosis are very similar. We found that larval neuroblasts from homozygous *endos* mutants show poorly condensed chromosomes and anaphase bridging, a phenotype very similar to recessive *gwl* mutants. In cultured *Drosophila* cells, depletion of *endos* interferes with proper mitotic exit and allows cells to accumulate that have elongated spindles but have not undertaken chromatid separation or Cyclin B destruction. This is similar to the removal of Gwl from CSF *Xenopus* extracts; there, an unusual mitotic exit occurs in which cyclins remained undegraded but Cyclin-dependent kinase 1 (Cdk1) is inactivated by phosphorylation at Thr14 and Tyr15 [3], [5].

**Figure 5:**
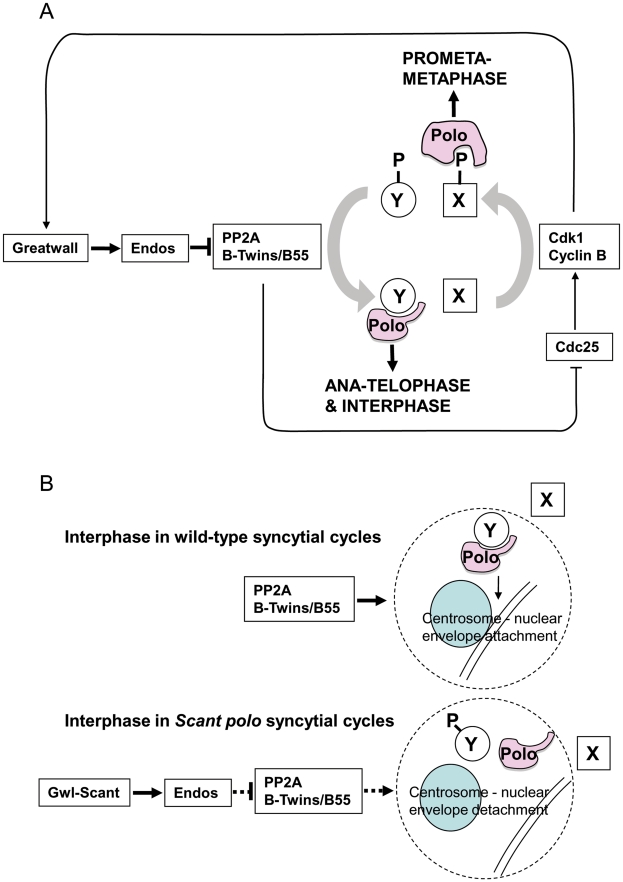
Model for PP2A function in mitotis and centrosome behaviour in concert with Greatwall and Polo. The gain-of-function *gwl^Scant^* phenotype is suppressed by partial loss of *endos* function and enhanced by partial loss of function of either the catalytic or Twins/B55 regulatory subunit of PP2A. Moreover, the mitotic defects of *endos* knockdown can be suppressed by knockdown of the catalytic or Twins/B55 regulatory subunit of PP2A but not the widerborst, B′ or B″ regulatory subunits. This together with biochemical data is in accord with a model whereby Greatwall phosphorylates Endos to convert it into an inhibitor of PP2A (specifically of the holoenzyme having the Twins/B55 subunit). PP2A is known to dephosphorylate Cdk1 substrates [11], [12], [25]. Thus inhibition of PP2A by phosphorylated Endos promotes the mitotic state when Cdk1 substrates are highly phosphorylated. The phenotype resulting from a gain-of-function allele of the Greatwall kinase is manifest only in the presence of reduced Polo levels, suggesting that Greatwall can negatively regulate Polo function. However, Polo function is also required for the mitotic state. (A) Our model accommodates the paradox and the enhancement of female sterility in *twins/polo^11^* transheterozygotes. This is because Polo binds to one set of targets that have been phosphorylated by Cdk1, exemplified by protein X in this diagram, and another set for which phospho-priming is not required, exemplified by protein Y [27]. Thus outside of true M-phase, PP2A promotes the dephosphorylation of Cdk1 substrates enabling Polo to bind to and function with its mitotic exit and interphase partners. (B) The loss of centrosomes from the nuclear envelope in embryos derived from *polo gwl^Scant^* mothers appears to reflect a tip in the balance of Polo's late mitotic/interphase functions in favour of its early mitotic functions. The function of Polo in regulating centrosome attachment to the nuclear envelope is seen as being positively regulated by PP2A. This phosphatase brings about dephosphorylation of protein Y enabling it to bind to Polo kinase to execute this function. Inappropriate activity of Greatwall^Scant^ leads to depression of PP2A activity *via* Endos and so reduces the interaction between Polo and protein Y. Conversely it increases Polo's interactions with protein X. Greatwall^Scant^ activity thus favours Polo's M phase functions and centrosome attachment to the nuclear envelope is particularly sensitive to this shift in the balance of Polo's functional activities.

Three lines of genetic evidence indicate that Greatwall and Endos are required to down-regulate the function of B55/Twins-bound PP2A. Lowering the dosage of either the catalytic C subunit or the B55/Twins regulatory subunit of PP2A enhances the maternal dominant effect of *polo^1^ gwl^Scant^* and this is suppressed by lowering the dosage of *endos*. Secondly, opposing roles for Endos and PP2A in regulating Polo kinase function are seen in the absence of the *gwl^Scant^* mutation; the low fertility of *twins/polo* trans-heterozygous females is also dramatically suppressed by one mutant copy of *endos*. Thirdly, the Endos depletion phenotype in cultured cells is suppressed by simultaneous depletion of either the catalytic C subunit, the structural A subunit, or the B55/Twins regulatory subunit of PP2A but notably not by co-depletion of the three other regulatory B subunits. Together these interactions suggest that Greatwall activates Endos leading to the inhibition of PP2A-B55/Twins. This is in accord with recent studies in *Xenopus* showing that inhibition or depletion of PP2A-B55 from mitotic extracts rescues the inability of Gwl-depleted extracts to enter M phase [6], [7] and also with two recent biochemical studies that show that the *Xenopus* counterpart of Gwl kinase can phosphophorylate two related members of the cAMP-regulated phosphoprotein family, Ensa (the Endos counterpart) or Arpp19, to make these molecules highly effective inhibitors of PP2A [13], [14]. Endos is the unique cAMP-regulated phosphoprotein family member in *Drosophila* [24]. Indeed, such is the degree of conservation that *Drosophila* Gwl kinase phosphorylates Endos only at Serine 68, a site essential for Endos function; this is the exact counterpart of the Serine 67 site in *Xenopus*. Studies in *Drosophila*, *Xenopus* and human cells [11], [12], [25] indicate that PP2A is a major protein phosphatase acting to dephosphorylate Cdk1 substrates. Thus *gwl* or *endos* reduced-function mutants should have increased activity of PP2A and therefore accumulate dephosphorylated Cdk1 substrates. Failure of Cdk1 substrates to become maximally phosphorylated in spite of high levels of Cyclin B accumulation would account for the prolonged prometaphase-like state and the eventual development of elongated spindles without having appeared to activate the anaphase-promoting complex in these mutants (Figure 5). This leads to a model in which Greatwall kinase is active in mitosis in order to convert Endos into an inhibitor of PP2A-Twins/B55, which is then inactived upon mitotic exit to permit the dephosphorylation of Cdk1 substrates by this phosphatase.

The above simple model is, however, confounded by genetic interactions suggesting that the gain-of-function mutation *gwl^Scant^* negatively regulates the function of the mitotic kinase Polo or one of its downstream targets. Such evidence comes largely from the search for suppressors of *polo^11^ gwl^Scant^* that identified mutations in two broad categories [4]: 1) those that decrease the effect of Gwl or its downstream targets as exemplified by *endos* mutations and reversion of *gwl^Scant^* to recessive mutant alleles; 2) those that increase the activity of Polo kinase such as the *polo^+^* duplications we obtained. Moreover, the degree of sterility (adult progeny per female) and frequency of embryonic centrosome loss co-vary with strength of *polo* allele [4]. *polo^1^*, a hypomorphic allele with sufficient residual Polo function to be homozygous viable, is slightly fertile heterozygous with *Scant* and its embryos are only moderately defective, whereas *polo^11^*, a lethal amorphic mutation, is completely sterile heterozygous with *Scant* and its embryos are much more defective. Furthermore, over-expressing Map205 (a known binding partner of Polo which sequesters the kinase on microtubules) in ovaries of *polo^11^/+* mothers mimics *Scant* regarding the centrosome detachment phenotype, and more defective nuclei are seen when the transgene carries a mutation preventing Polo release [26].

Together our results suggest that the specific defect in *Scant polo*-derived embryos, detachment of centrosomes from the nuclear envelope, is a consequence of the reduction of the level of functional Polo below a critical threshold. Indeed this is the only phenotype we have been able to attribute to the *Scant* allele of *gwl* and its sensitivity to the gene dosage of *polo* suggests that this function requires the highest level of Polo kinase activity in comparison to all of Polo's other roles. It is important to note that centrosome detachment is an interphase phenotype. It occurs after the centrosomes have separated, which in wild type is during telophase in anticipation of the next round of mitosis in the rapidly alternating S and M phases of the syncytial *Drosophila* embryo. In the normal mitotic cycle, Greatwall kinase would not be active at this stage. Thus the functional complex of PP2A and its B55/Twins regulatory subunit seems to be required to positively regulate Polo activity or a process controlled by Polo between the exit from one mitotic cycle and entry into the next. This accounts for our finding that mutations in the PP2A subunit genes, *mts* and *twins*, enhance sterility when transheterozygous with *polo^11^*, and that this sterility is in turn relieved by heterozygous *endos* mutations. Although it is possible that PP2A removes an inhibitory phosphorylation from Polo, this seems unlikely because no such phosphorylation has been identified to date. Thus we favour the alternative that PP2A acts to stimulate a process promoted by Polo and a dephosphorylated partner. Indeed it is known that Polo interacts with phosphorylated partners after mitotic entry and with dephosphorylated partners from late anaphase onwards (reviewed in [27]).

We depict this model in the context of the oscillating Cdk1+ (prophase and prometaphase) *vs.* Cdk1- (post anaphase) states in Figure 5A. In prophase and prometaphase Greatwall, activated by Cdk1, inhibits PP2A *via* Endos. This sustains the Cdk1 phosphorylation that enables Polo to bind phosphorylated partners (*e.g.*, protein X in Figure 5A) for its early mitotic functions. Once Cdk1 activity levels have fallen at anaphase, Polo instead binds a set of proteins dephosphorylated at their Cdk1 sites by PP2A-B55/Twins (*e.g.*, protein Y in Figure 5A). At least one such protein of the Y-type is required to maintain attachment of the centrosome to the nuclear envelope although the specific functions of Polo in maintaining the attachment of the centrosome to the nuclear envelope remain to be uncovered. We suggest that, in the syncytial embryo, the kinase-activating *Scant* mutation of Greatwall leads to inappropriate inactivity of PP2A in interphase reducing the levels of functional complexes between Polo and dephosphorylated partners (Figure 5B). In this model, the gain-of-function Greatwall^Scant^ kinase tips the balance by transiently reducing interphase PP2A levels to a point that prevents sufficient dephosphorylation of Polo's interphase partner(s). We postulate that one such partner might be required for a threshold activity of Polo needed to maintain centrosome attachment.

Supporting evidence for the combined roles of PP2A and Polo in a common process comes from the accompanying study [28]. In this analysis, Archambault's lab has systematically placed chromosomes carrying either the *polo^11^* allele or *gwl^Scant^* against chromosome deficiencies from the DrosDel deficiency core kit. This led to the independent identification of *twins* as an enhancer of *polo^11^* and of *gwl^Scant^*; as in our study, embryos derived from *twins/polo^11^* or *twins/gwl^Scant^* trans-heterozygous mothers exhibit lethality. Interestingly, their analysis of the mutant phenotype of both *polo^11^* or *twins-*derived embryos indicated that both genes were indeed required to maintain the attachment of centrosomes to the nuclear envelope.

Our studies support a major mitotic role of the Greatwall kinase in phosphorylating Endos to activate it as an inhibitor of PP2A that is bound to its B55/Twins regulatory subunit and thus promote the mitotic state. This model can accommodate a differential effect upon Polo kinase regulation depending upon the phase of mitosis and so seems likely to be a conserved central tenet of mitotic regulation. However, there are other anomalies that suggest that this may not be the complete story. Several pieces of evidence point to PP2A not being the only protein phosphatase able to reverse Cdk1-mediated phosphorylation. A debate about the relative importance of PP1 and PP2A in this function has been ongoing for years and indeed PP2A in association with other regulatory B subunits has other mitotic functions [29], [30]. We were unable to demonstrate a substantial inhibitory effect of phospho-Endos upon the ability of PP2A to dephosphorylate histone H1 phosphorylated by Cdk1 (data not shown). Gharbi-Ayachi and colleagues and Mochida and colleagues, using respectively either c-Mos or a phosphopeptide derived from Cdc20, were able to demonstrate such an inhibitory activity with *Xenopus* phospho-Endos [13], [14] although the use of other substrates for this type of assay was apparently less effective (Hunt, personal communication). The involvement of other phosphatases would certainly add complexity to this simple model.

From a genetic perspective, it is also noteworthy that we observed only weak zygotic interactions between *endos* and *twins* mutants. For example, adding either *tws* allele to either *endos* allele (as *endos+/endos tws*) rescues the eclosion delay and the mild cuticular phenotype but not the female sterility; since the delays are only 1 or 2 days in the first place, this rescue is mild, though it is encouraging that the apparently stronger *endos^EYO1105^* is rescued just as well as the apparently weaker *endos^EYO1103^* allele. Although none of these mutations is amorphic, they nevertheless each reduce their products enough so that even as heterozygotes they give strong interactions in the *polo^1^ Scant* test.

The above considerations, together with the finding that *endos* null mutants (*e. g., endos^1^*) are viable (but sterile), suggests that Endos function may have particular importance in the germline and in the rapid cycles of the early embryo. In this context, it is important to distinguish between the effects we describe here of *endos* on Polo function and previously reported effects of *endos* on Polo levels in the female germline [16]. The present study examines embryos derived from heterozygous females that have at least 50% of the wild-type levels of Endos and in which levels of Polo are not significantly affected. However, this reduction in Endos is sufficient to rescue a common function of *polo* and *PP2A-twins* in the nuclear cycles of the syncytial embryo. In this context, the function of wild-type Endos levels would be to down-regulate Polo activity. On the other hand, the study of Von Stetina and colleagues [16] examines female meiosis in heteroallelic or hemizygous *endos* combinations where levels of Endos are reduced by more than 95%. This leads to substantial reductions in Polo kinase levels that are likely to contribute significantly to the meiotic phenotypes observed. In this context, the requirement for Endos appears to be to facilitate the post-transcriptional regulation of the expression of the *polo* (and *cdc25^twine^*) genes. This might have resonance in the recently demonstrated functions of budding yeast proteins Rim15, Igo1 and Igo2, (counterparts of Greatwall, Endos/Ensa and Arpp19) in translational control in quiescent cells [31]. The observations that Polo protein is reduced in oocytes/ovaries of *endos* homozygotes ([16], Figure S1) but not in ovaries of heterozygotes (Figure S1) and apparently not in their embryos (*endos/polo* and *endos/gwl^Scant^* females are fertile) or in cultured cells after Endos depletion suggest that female meiosis and early embryogenesis have different regulation. Indeed, this could reflect the absence of centrosomes in female meiosis. Finally, it is unlikely that Endos is the only substrate of Greatwall kinase. This may help to provide an explanation for the observation that, although down-regulation of *gwl* or *endos* function leads to similar defects in mitosis, the phenotypes of their mutants in female meiosis are quite different. *endos* mutants show prolonged prophase and failure to progress to metaphase I, whereas a germline-specific *gwl* mutant exhibits failure to maintain arrest at metaphase I and premature progression through all of the meiotic stages. Taken together, these anomalies are reminders that our understanding of the regulation of protein phosphatases in cell cycle progression is still rudimentary.

## Materials and Methods

### Fly stocks and procedures

Fly stocks used were described previously [4], [20] or obtained from the Bloomington *Drosophila* Stock Center. The *endos* alleles *79*, *60*, and *1* resulted from remobilisation of the P element in *P{lacW}endos^SO67006^*, referred to as *endos^67006^* in this paper. The *endos* alleles *EY01103* and *EY01105* are also associated with P-element insertions (FlyBase); both strongly reduce the level of protein detected by an anti-Endos antiserum in Western blots (Figure S1) though traces remain.

Female fertility was tested by taking single newly-eclosed females, adding Oregon R males, and scoring daily for the onset of egg laying; that day = day 0. Parents were transferred to fresh food every three days for a total of 15 days egg laying then discarded; progeny were counted (each vial separately) until eclosion was complete. Six females per genotype were tested, three each on two types of medium; the results presented here are all from the richer medium – the other medium gave identical rankings.

Relative strengths of the *endos* P insertion alleles *EY01103* and *EY01105* and the *tws* alleles *aar1* and *P* are based on phenotypic observations, namely: *EY01105* has longer eclosion (adult emergence from the pupal case) delay than *EY01103*, so it seems to be the stronger of the two, even though the levels of residual protein in ovary extracts are indistinguishable (Figure S1A). Moreover, *EY01103* homozygous males are fertile, so it is a hypomorphic mutation (see text *re endos^79^*). Males homozygous for the *EY01105*-bearing chromosome are sterile, but this sterility has not been mapped so may be due to an additional, extraneous male sterile mutation. *aar1/P* pupae die later than *P/P* so *P* seems to be the stronger. These rankings are consistent with other phenotypic comparisons, see Results.

### DNA constructs

Gateway vector pAGW from Invitrogen encoding GFP downstream of the *actin 5C* promoter and *greatwall* Gateway entry clones [4] were used as plasmid templates to generate dsRNA directed against GFP and Greatwall respectively.

The *endos* entry clones were generated according to the instructions for the Gateway system (primers are listed separately, Table S4). *endos* (*CG6513*) cDNA was obtained from DRGC clone LD18034 (http://dgrc.cgb.indiana.edu/). PCR products for the *endos* ORF flanked with *att* recombination sites were generated from LD18034 and ligated into the pDONR221 entry vector. Entry clones of *endos*, pDONR221-*endos*-stop and pDONR221-thrombin-*endos*-stop were generated that either did or did not contain a cleavage site for thrombin.

The *endos* S68A and S68D mutants were made in the entry clones with the quick-change Site-Directed Mutagenesis Kit (Stratagene, see primers list in Table S4).

GST-*endos* and GST-*endos* S68A constructs were obtained by LR recombination of pDONR221-thrombin-*endos*-stop and pDONR221-thrombin-*endos* S68A-stop entry clones into pDEST15 Gateway destination vector.

pActin-HA-*endos*, pActin HA-*endos* S68A and HA-*endos* S68D constructs were obtained by LR recombination of pDONR221-*endos*-stop, pDONR221-*endos* S68A-stop and pDONR221-*endos* S68D-stop entry clones into the pAHW Gateway destination vector.

The CG6513 (*endos*) rescue construct was generated by PCR amplification from fly genomic DNA with primers flanked with NotI restriction sites and cloned into the pCasPer4 vector using its NotI restriction site (primers are listed in Table S4).

The CG6650 (divergent transcript) rescue construct was generated by PCR amplification from fly genomic DNA with primers flanked with NotI restriction sites and cloned into the pCasPer4 vector using its NotI restriction site (primers are listed in Table S4).

The pCasPer4-*endos*-EGFP construct was generated from the CG6513 rescue construct by inserting an EGFP cassette in frame just before the stop codon of *endos*. A BglII restriction site was created by site-directed mutagenesis in place of the stop codon of *endos* in the CG6513 rescue construct so that the EGFP cassette flanked by BglII restriction sites could be inserted there (primers are listed in Table S4).

### Antibodies

The following antibodies were used: rabbit anti-phospho-Histone H3 (Upstate), immunofluorescence (IF) 1/4000 for cells, 1/500 for larval brains; chicken anti-Dplp [32], IF 1/1000; mouse anti-α-tubulin (DM1A, affinity purified, Sigma), IF 1/1000, Western blot (WB) 1/10000; rabbit anti-GFP (Molecular Probes), IF 1/600; rabbit anti-actin (A2066, Sigma), WB 1/2000; mouse anti-γ-tubulin (GTU88, Sigma); chicken anti-Cid [33] (10811), IF 1/2000; rabbit anti-Cyclin B (Rb271) [34], IF 1/200; anti-Mei-S332 [35], IF 1/10000; rabbit anti-Endos (7648), WB 1/3000. The antibody specific to Endos phosphorylated at Serine 68 was a generous gift from T. Hunt and S. Mochida [13]. This antibody was initially raised against the sequence surrounding the phosphorylated Ser67 in the *Xenopus* counterpart of Endos, Ensa : QKYFDSpGDYN. This sequence is close to the sequence surrounding the Ser68 in *Drosophila* Endos : QKFFDSpGDYQ.

The secondary antibodies used were conjugated with Alexa 488, Alexa 568 or Alexa 697 (Molecular Probes, 1/800) and peroxidase (Jackson Immunochemicals, 1/10000).

### Cell culture

DMEL cells were grown at 25°C in Express Five SFM *Drosophila* media (Invitrogen) supplemented with L-glutamine (2 mM, Gibco) and penicillin-streptomycin (50000units/L-50000 µg/L, Gibco).

DMEL cells stably expressing Myc-Gwl wt, Myc-Gwl K87R (kinase dead form) and Myc-Gwl K97M (hyperactive form) under the actin promoter were described in [4]. DMEL cells stably expressing GFP-Cid and β-tubulin-mRFP were the generous gift of Luisa Capalbo (University of Cambridge, Department of Genetics).

Okadaic acid (Potassium salt, Calbiochem) treatments were performed by adding the drug at the indicated final concentration for 2 h prior to harvesting the cells.

### dsRNA experiments

dsRNAs against *endos* (coding region), *greatwall* and *GFP* (as control) were each made from plasmid DNA. dsRNAs against the 3′UTR of *endos* and the PP2A subunit genes *microtubule star*, *29B*, *twins*, *widerborst*, *B′* and *B″* were all made from genomic DNA generated from DMEL cells. A list of primer pairs is given in the primers list (Table S4). 1.4×10^6^ cells per well were plated in 6-well plates one day before transfection with 25 µg of dsRNA. For co-depletions, 25 µg of each dsRNA was used and single dsRNAs were supplemented with 25 µg of control dsRNA. dsRNAs in 10 µl of H_2_O were incubated with 20 µl of Transfast (Promega) and 970 µl of medium (960 µl for co-depletions) for 15 min before transfection. The dsRNA solution (1 ml mix) was then incubated on the cells for 1 h prior to the addition of 3 ml of medium. Cells were harvested after 3 days.

### Immunofluorescence and fluorescence microscopy

Brains from 3^rd^ instar larvae were dissected in PBS, fixed 20 min in PBS containing 10% formaldehyde, permeabilised in PBST (PBS-0.1% Tween 20) for 2 min, and preincubated in PBST containing 1% BSA. Overnight incubations with the primary antibody were followed by 2×20 min washes before incubation for 2 h with secondary antibodies. Brains were washed again before mounting on slides in Vectashield containing DAPI (Vector Laboratories).

DMEL cells were harvested and plated on 13 mm diameter glass coverslips coated with concanavalin-A in a 24-well plate at 3×10^5^ cells per well for 1–2 h before fixation. Cells were then pre-extracted for 5 s in 0.1% NP40 in BRB80 buffer (80 mM K-Pipes pH 6.8, 1 mM MgCl_2_, 1 mM Na-EDTA pH 8) and immediately fixed in BRB80-4% formaldehyde for 20 min. They were then permeabilised in BRB80-0.1% Triton-X100 for 10 min and washed 3×5 min with PBS. Antibodies were diluted in PBS containing 0.1% Tween 20-3% BSA and incubated for 1 h at room temperature or overnight at 4°C for primary antibodies and for 1 h at room temperature for secondary antibodies. Samples were washed after each incubation in PBS-0.1% Tween 20. Finally, cells were rinsed in water and mounted on slides in Vectashield containing DAPI (Vector Laboratories).

Images were acquired with a Zeiss Axiovert 200 M microscope using a 100× objective, 1.4 NA, and a Coolsnap HQ2 camera controlled by Metamorph software (Universal Imaging). Figures shown are projections of optical sections acquired at 0.2 µm z steps. Some image stacks were deconvolved using 10 iterations of the blind deconvolution algorithm in the AutoquantX software (Media Cybernetics). All images were imported into Adobe Photoshop for contrast manipulation and figure assembly.

For analysis, mitotic cells were detected by their phospho-Histone H3 staining. The phenotype of dispersed chromosomes in prometaphase was quantified in cells whose DAPI-stained chromosomes are not aligned on the equatorial plate. Cells exhibiting at least one chromosome (disregarding the small 4^th^ chromosome) near the spindle poles (visualized with Dplp as a centrosomal marker) were scored as defective. This phenotype is given relative to the number of cells in prometaphase. Telophases with lagging chromosomes/chromosome bridges were quantified in cells whose two daughter nuclei had reformed. Cells exhibiting chromosomes dispersed between the two re-forming nuclei were scored as defective. This phenotype is relative to the number of cells in telophase.

### Time lapse analysis of *Drosophila* syncytial embryos and DMEL cells

Flies expressing Endos-EGFP were held in egg-collection chambers and embryos were collected after 1 h at 20°C. Embryos were transferred to a sieve, dechorionated in 50% bleach for 1 to 2 min and washed with water. They were transferred to a drop of Voltalef oil on a membrane maintained in a metal frame and a 22×40 mm coverslip was placed on top.

DMEL cells stably expressing GFP-Cid and β-tubulin-mRFP were depleted for Endos for three days or left untreated (control).

Time lapse recordings were carried out using a Zeiss Axiovert 200 fluorescence microscope equipped with the Perkin Elmer UltraVIEW RS confocal scanner and Volocity software. Images of embryos were acquired with a 63× objective, NA 1.4, at a *z*-distance of 1 µm between planes using 2× 2 binning, every 30 s. Images of DMEL cells were acquired with a 100× objective, NA 1.4, using 10 z slices and ca. 1 µm between planes with 2× 2 binning. Images were taken every 2 min for control cells and every 5 min for cells after Endos depletion.

### Flow cytometric analysis of cell DNA content

1 ml of cells depleted for GFP or Endos for 3 days were pelleted at 1000 rpm for 5 min and resuspended with 200 µl PBS. 2 ml of cold 70% ethanol was added dropwise to the resuspended cells while vortexing. Cells were stored at −20°C until analysis. Before analysis, 10 ml of PBS was added to the cells and they were pelleted at 1800 rpm for 10 min. The supernatant was carefully removed and the cells were resuspended in 0.5 ml PBS containing 100 µg/ml propidium iodide (Sigma) and 100 µg/ml RNAse A (from bovine pancreas, Sigma) and incubated for 30 min at 37°C. The DNA content of the cells was analysed using a Beckton Dickinson FACScan and LSR, which required 30,000 cells for each sample. Results were analysed with Summit software from DaKoCytomation.

### Preparation of protein extracts and Western blot analysis

Protein extracts from tissue-culture cells were prepared by resuspending pellets of cells in SDS-PAGE sample buffer (100 µl of sample buffer 2× per 1×10^6^ cells) and boiling for 5 min. Extracts equivalent to 6×10^5^ cells were processed for Western blot analysis.

Brains from third instar larvae were dissected in PBS and kept at −80°C. Tissues were pestle homogenized in 1D lysis buffer (50 mM Tris pH 8, 150 mM NaCl and 1% NP40; 5 µl of buffer per brain), incubated on ice for 20 min and centrifuged at 13,000 rpm for 10 min. Soluble protein fractions were processed for Western blot analysis; variable amounts of samples were used to obtain equal loading: Oregon R, *endos^67006^*, CG6513, CG6650, *endos^79^*, each equivalent to 1 brain; *endos^60^*, equivalent to 1½ brains; *endos^1^*, equivalent to 2 brains.

Ovaries of adult flies were dissected in 0.7% NaCl and kept on dry ice until preparation of the extract. Tissues were pestle homogenized in 1D lysis buffer (5 µl of buffer per pair of ovaries), incubated on ice for 20 min and centrifuged at 13,000 rpm for 10 min. The protein concentration of soluble protein fractions was quantified and 25 µg of proteins were processed for Western blot analysis.

Cell, larval brain extracts or ovary extracts were loaded onto SDS-PAGE (ProGel Tris Glycine 8–16%, Anamed) and transferred onto nitrocellulose membranes (Hybond ECL, Amersham Biosciences). Membranes were blocked with TBS-0.2% Tween 20-3% BSA for 30 min at room temperature. Incubation with primary or secondary antibodies diluted into TBS-0.2% Tween 20-3% BSA were performed at 4°C overnight or 1 h at room temperature respectively. Peroxidase activity was detected with the Amersham ECL plus Western blotting detection system (GE healthcare).

Phos-tag containing gels were prepared by adding 25 µM of Phos-tag reagent (Wako) and 50 µM of MnCl_2_ to the mix used to prepare a 12% Tris Glycine SDS-PAGE. Gels were incubated 10 min in running buffer containing 1 mM EDTA and then 10 more minutes in running buffer only to wash out the EDTA before transfer onto nitrocellulose membranes. Transfers were performed using the Iblot Dry Blotting Transfer system from Invitrogen (9 min transfer).

### Transient transfections of *endos* constructs into DMEL cells

Transient transfections were performed to assess the effect of over-expression of wild-type or the S68A and S68D forms of *endos* in cells previously treated for 24 h with dsRNA against GFP or the 3′UTR of endogenous *endos*. 2 ml of the 4 ml of culture medium was removed before transfection. 3 µg of HA-tagged *endos* constructs was diluted in 100 µl of H_2_O. 15 µl of Fugene-HD (Roche) was mixed with the DNA and incubated at room temperature for 15 min. 115 µl of the mix was then added to the cells. 2 days after transfection (and 3 days after depletion), the cells were harvested and processed for immunofluorescence or Western blot analysis.

### Production of recombinant Endos in bacteria

GST-tagged Endos and GST-tagged Endos (S68A) proteins were produced in BL21 Star pLysS bacteria (Invitrogen) after IPTG induction. Soluble proteins were purified with glutathione-Sepharose-coupled beads (GE Healthcare) and Endos and Endos (S68A) were dissociated from beads by cleavage with Thrombin protease (Amersham Biosciences) for 2 h at room temperature according to the manufacturer's instructions.

### Phosphorylation assay

Immunoprecipitation of Myc-tagged Greatwall kinases and phosphorylation assays were performed as described previously [4]. Briefly, Myc-tagged Greatwall wild-type, K87R and K97M were immunoprecipitated from DMEL cell lines stably expressing the kinases. A solution of PBS containing thrombin protease at the same dilution as the one in which Endos substrates are kept was used as the negative control. Kinases and substrates were incubated for 30 min at 30°C with γ-^32^P-ATP. The reaction products were resolved by SDS-PAGE and stained with Coomassie Blue (Bio-Safe Coomassie G-250 stain, BIO-RAD); ^32^P-labeled proteins were visualized by autoradiography.

### Mass spectrometry

Peptide mixtures were analyzed by liquid chromatography coupled to Orbitrap Velos mass spectrometry (Hybrid-2D-Linear Quadrupole Ion Trap – Orbitrap Analyzer Mass Spectrometer, Thermo Electron Corp.). Prior to the analysis, gel slices were subjected to a standard “in-gel digestion” procedure during which proteins were reduced with 100 mM DTT (for 30 min at 56°C), alkylated with iodoacetamide (45 min in a darkroom at room temperature) and digested overnight with trypsin (sequencing Grade Modified Trypsin, Promega). The resulting peptides were eluted from the gel with 0.1% TFA, 2% ACN. The peptide mixture was applied to an RP-18 precolumn (nanoACQUITY Symmetry C18, Waters) using water containing 0.1% TFA as mobile phase and then transferred to a nano-HPLC RP-18 column (nanoACQUITY BEH C18, Waters) using an acetonitrile gradient (0%–60% AcN in 30 min) in the presence of 0.05% formic acid with a flowrate of 150 nl/min. The column outlet was directly coupled to the ion source of an LTQ-Orbitrap-Velos-MS working in the regime of data-dependent MS to MS/MS switch. A blank run to ensure lack of cross contamination from previous samples preceded each analysis.

Acquired raw data were processed by Mascot Distiller followed by Mascot Search (Matrix Science, locally installed http://proteom.pl/mascot) against FlyBase. Search parameters for precursor and product ion's mass tolerance were respectively ±20 ppm and ±0.4 Da, without allowance for missed Trypsin cleavage sites, fixed modifications of Cysteine through carbamidomethylation or variable modifications through Lysine carbamidomethylation, Methionine oxidation, Serine, Threonine and Tyrosine phosphorylation. Proteins of interest were then subsequently submitted for an Error Tolerant search with enzyme specificity changed into semiTrypsin and allowance for one missed semiTrypsin cleavage site. Peptides with Mascot Score above the expectation cut-off (for FlyBase and the above Mascot search parameters, the threshold was set at 21) were considered to be significant. Presence of phosphorylation was confirmed on the basis of visual inspection of the spectra.

## Supporting Information

Figure S1 Alleles of *endos*. A. Analysis of Endos and Polo levels in ovaries of wild-type (*Oregon-R*) flies and of *endos^EY01103^* or *endos^EY01105^* mutant flies. Ovary extracts from females either heterozygous (*endos*/*balancer*) or homozygous for *endos* were blotted against antibodies detecting Endos, Polo and Actin (loading control). Weak bands are detected by an anti-Endos antiserum in extracts of homozygous flies showing a strong but not complete depletion of Endos. The levels of Polo protein are significantly reduced in homozygous flies but not in heterozygotes, suggesting a correlation between Endos and Polo levels. B. Gene region showing the location of the P element in *endos^67006^*and detailing the additional alleles *endos^79^*, *endos^60^* and *endos^1^* generated during the study (these alleles were used in the analysis of larval neuroblast phenotypes). The predicted gene model is shown in blue and the coding sequences are shown in red; modified from FlyBase. A, B, C and D indicate primers used to generate genomic rescue constructs by PCR. The black bars show the regions of genomic DNA that have been deleted in the alleles *endos^60^* and *endos^1^*. C. Protein levels of Endos in wild type (*Oregon-R*) and the indicated *endos* genotypes. Extracts prepared from larval central nervous system were analysed with antibodies detecting Endos and Actin (loading control). Endos protein levels are rescued by an *endos^+^* transgene but not by a *CG6650* transgene. The alleles generated during the study lead to various levels of expression of Endos protein.
(TIF)

Figure S2 Knockdown of Endos by RNAi in DMEL cells. A. Flow cytometric analysis following *endos* RNAi shows a decreased G1 peak relative to the G2/M peak. B. Depletion of Endos protein after *endos* RNAi treatment but not control RNAi treatment (*GFP*). Actin is the loading control.
(TIF)

Figure S3 Localisation of Endos in syncytial embryos and cultured DMEL cells. A. In flies transgenic for *endos-EGFP*, green fluorescence is seen mainly within the area bounded by the spindle envelope and also accumulates around centrosomes (arrow in panel 7.0 minutes). Endos-EGFP fluorescence intensity increases prior to the formation of the spindle (compare panels 0, 1.5 and 3.0 minutes), remains high throughout metaphase (panels 4.0 to 7.0 minutes), but appears to decrease after anaphase (compare panel 8.0 and 10.0 minutes) and remains low until the next mitotic division. Note the low levels of cytoplasmic Endos-EGFP throughout division. Time is indicated in minutes. Scale bar represents 10 µm. B. In DMEL cells expressing *endos-EGFP*, fluorescence is present both in the nucleus and around the centrosomes in prophase cells (panel a). It localises to the spindle region at metaphase (panel b) and its level decreases following anaphase (panel c; note that it is still associated with the central spindle). Endos-EGFP is also present at lower levels in the cytoplasm throughout mitosis. Cells are stained to reveal microtubules in red, Endos-GFP in green, and DNA in blue. Scale bar represents 10 µm.
(TIF)

Figure S4 Phenotypes of DMEL cells after Endos and PP2A combined depletion. A. Depletion of Endos, alone or in combination with PP2A subunits, minimally affects levels of Polo kinase at best. Levels of proteins are visualised by Western blot after single or double depletion of DMEL cells for GFP (control depletion), Greatwall (Gwl), Endos (End) and the various subunits of PP2A: Microtubule Star (Mts), 29B, Twins (Tws) or Widerborst (Wdb). Extracts were analysed with antibodies detecting Polo and Actin (loading control). B. Additional phenotypes after depletion of Endos and PP2A subunits. After double depletion of Endos and either the catalytic subunit (Mts) or the structural subunit (PP2A 29B) of PP2A, (pro)metaphases show some long microtubules that are not captured by the spindle and may or may not be astral microtubules. Scale bar represents 5 µm. C. Lagging chromosomes and chromosome bridges after *endos* depletion are suppressed by PP2A-*twins*/B55 knockdown. Mitotic defects following Endos RNAi treatment were scored as proportions of telophases with lagging chromosomes and/or chromosome bridges. The *endos* knockdown phenotype is suppressed when the PP2A-*twins* subunit is also knocked down. Error bars represent sem of three independent experiments. P values are from a Student's T-test with *** = p<0.001; a minimum of 200 telophases were scored per treatment. D. Representative mitotic figures for the above depletions as indicated. Scale bar represents 5 µm.
(TIF)

Figure S5 Phosphorylation of Endos. A. Proteins used in the phosphorylation assay. Kinases immunoprecipitated from cell extracts (Myc-Greatwall wild type (Gwl wt), Myc-Greatwall kinase dead (Gwl KD, mutated for Lys87Arg) or Myc-Greatwall hyperactive form (Gwl act., mutated for Lys97Met)) and Endos substrates (wild type (Endos wt) or mutated for Ser68Ala (Endos S68A)) were resolved independently on SDS-PAGE. The gel was stained with Coomassie Blue (left panel), and the ^32^P-labeled proteins were visualised by autoradiography (right panel). None of the proteins used in the phosphorylation assay become labelled by ^32^P as detected by autoradiography indicating the absence of non-specific phosphorylation. Levels of Greatwall immunoprecipitated were also analysed by Western blot (data not shown and published in [4]). B. Endos is phosphorylated at Ser68 in cells and phosphorylation is enhanced by treatment with okadaic acid. DMEL cells were treated with none or the indicated concentrations of okadaic acid for 2 hours before preparation of cells extracts. The extracts were analysed on independent Western blots using antibodies detecting either Endos phosphorylated at Ser68 (upper panels) or Endos (lower panels) and tubulin (loading control). Phosphorylation of Endos at Ser68 is observed in cells and is increased after treatment with 25 nM okadaic acid (B upper panel). Such okadaic acid treatment also leads to an increase of the total Endos level in cells (B lower panel). C. Endos is phosphorylated at Ser68 in addition to other amino acid residues in cells. The same extracts as described in B were analysed on SDS-PAGE gels in the presence of Phos-tag instead of regular SDS-PAGE gels. The shift in Endos mobility that is seen after treatment with 5 nM okadaic acid suggests a block to dephosphorylation by PP2A. This particular modification is not recognised by the anti-P-Ser68 antibody. This antibody does, however, recognise a form of Endos that appears after treatment with 25 nM okadaic acid, indicating that the P-Ser68 modification is preserved by inhibiting a protein phosphatase other than PP2A. Treatment with 100 nM okadaic acid reveals a further band shift suggesting at least a third phosphorylation site sensitive to yet another protein phosphatase.
(TIF)

Table S1 Mitotic defects in *endos* mutants. Mitosis in cells of fixed preparations of larval central nervous systems of the indicated genotype were analysed according to the following criteria: mitotic index (MI), Metaphase∶Anaphase (M∶A) ratio and percentage of Anaphase defects. The deficiency indicated *Df* corresponds to the deficiency *Df(3L)fz-GF3b*. Extreme chromosome bridging made it difficult to recognise anaphase cells in *endos^67006^/Df(3L)fz-GF3b* and *CG6650/CyO*; *endos^67006^/Df(3L)fz-GF3b* and could account for the very high assessment of the Metaphase∶Anaphase ratio in these lines.
(DOC)

Table S2 Quantitation of mitotic defects following *endos* RNAi. Cultured cells depleted for Endos or GFP (control) were analysed for several mitotic parameters: mitotic index (MI), percentage of spindle defects, percentage of chromosome defects and the relative percentage of Telophase/Cytokinesis cells. A total of 281,363 GFP-depleted cells were scored of which 9,506 were in mitosis. A total of 240,442 Endos depleted cells were scored of which 18,688 were in mitosis.
(DOC)

Table S3 Details of DMEL cells expressing GFP-Cid and β-tubulin-mRFP analysed by time lapse imaging. Cells expressing GFP-Cid and β-tubulin-mRFP were treated with *endos* dsRNA or untreated and analysed by time lapse imaging. The table reports the duration of the prometaphase for each cell recorded. The table also indicates whether nuclear envelope breakdown and anaphase could be observed during the duration of the recording.
(DOC)

Table S4 Primers list. Summary of the primers used during this work. Note that dsRNA primers directed against subunits of PP2A are identical to the ones published in [33].
(DOC)

Video S1 DMEL expressing GFP-Cid and β-tubulin-mRFP (control for Video S2).
(AVI)

Video S2 DMEL expressing GFP-Cid and β-tubulin-mRFP and depleted for Endos.
(AVI)
