# Supplementary figures and images for: Suppression of Scant Identifies Endos as a Substrate of Greatwall Kinase and a Negative Regulator of Protein Phosphatase 2A in Mitosis
Source: PLoS Genet. 2011 Aug 11;7(8):e1002225. doi: 10.1371/journal.pgen.1002225 (PMC3154957; doi:10.1371/journal.pgen.1002225)

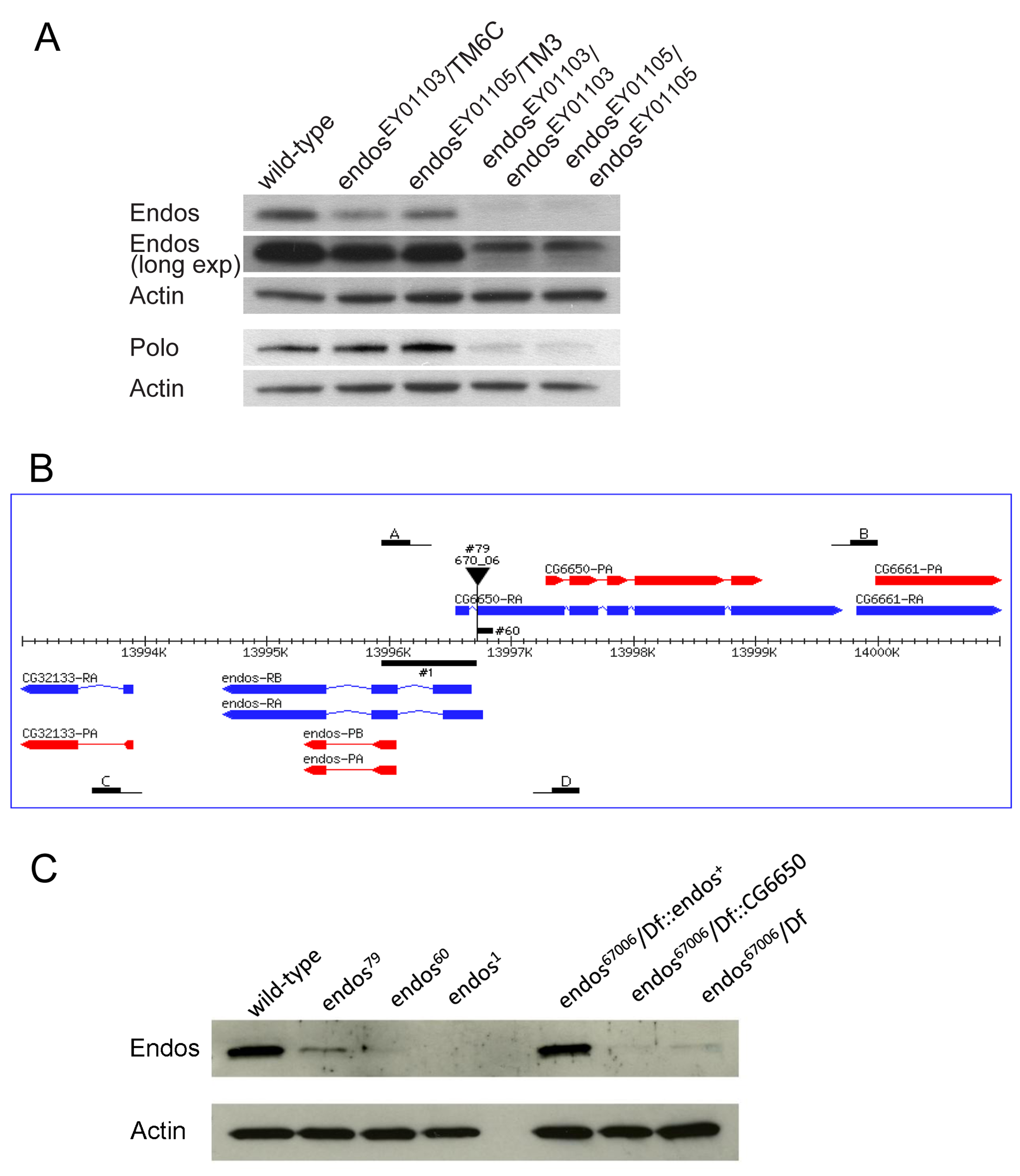

Supplement: Figure S1 — Alleles of endos. A. Analysis of Endos and Polo levels in ovaries of wild-type (Oregon-R) flies and of endosEY01103 or endosEY01105 mutant flies. Ovary extracts from females either heterozygous (endos/balancer) or homozygous for endos were blotted against antibodies detecting Endos, Polo and Actin (loading control). Weak bands are detected by an anti-Endos antiserum in extracts of homozygous flies showing a strong but not complete depletion of Endos. The levels of Polo protein are significantly reduced in homozygous flies but not in heterozygotes, suggesting a correlation between Endos and Polo levels. B. Gene region showing the location of the P element in endos67006and detailing the additional alleles endos79, endos60 and endos1 generated during the study (these alleles were used in the analysis of larval neuroblast phenotypes). The predicted gene model is shown in blue and the coding sequences are shown in red; modified from FlyBase. A, B, C and D indicate primers used to generate genomic rescue constructs by PCR. The black bars show the regions of genomic DNA that have been deleted in the alleles endos60 and endos1. C. Protein levels of Endos in wild type (Oregon-R) and the indicated endos genotypes. Extracts prepared from larval central nervous system were analysed with antibodies detecting Endos and Actin (loading control). Endos protein levels are rescued by an endos+ transgene but not by a CG6650 transgene. The alleles generated during the study lead to various levels of expression of Endos protein. (TIF) [file pgen.1002225.s001.tif]

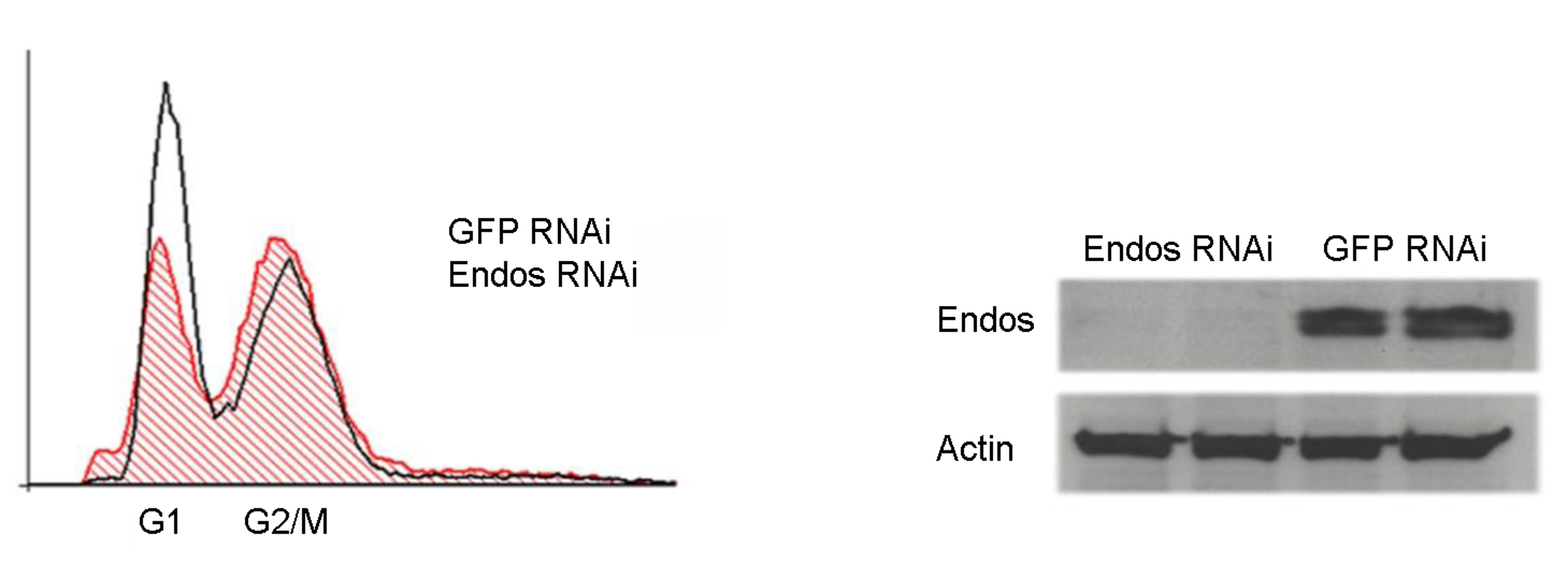

Supplement: Figure S2 — Knockdown of Endos by RNAi in DMEL cells. A. Flow cytometric analysis following endos RNAi shows a decreased G1 peak relative to the G2/M peak. B. Depletion of Endos protein after endos RNAi treatment but not control RNAi treatment (GFP). Actin is the loading control. (TIF) [file pgen.1002225.s002.tif]

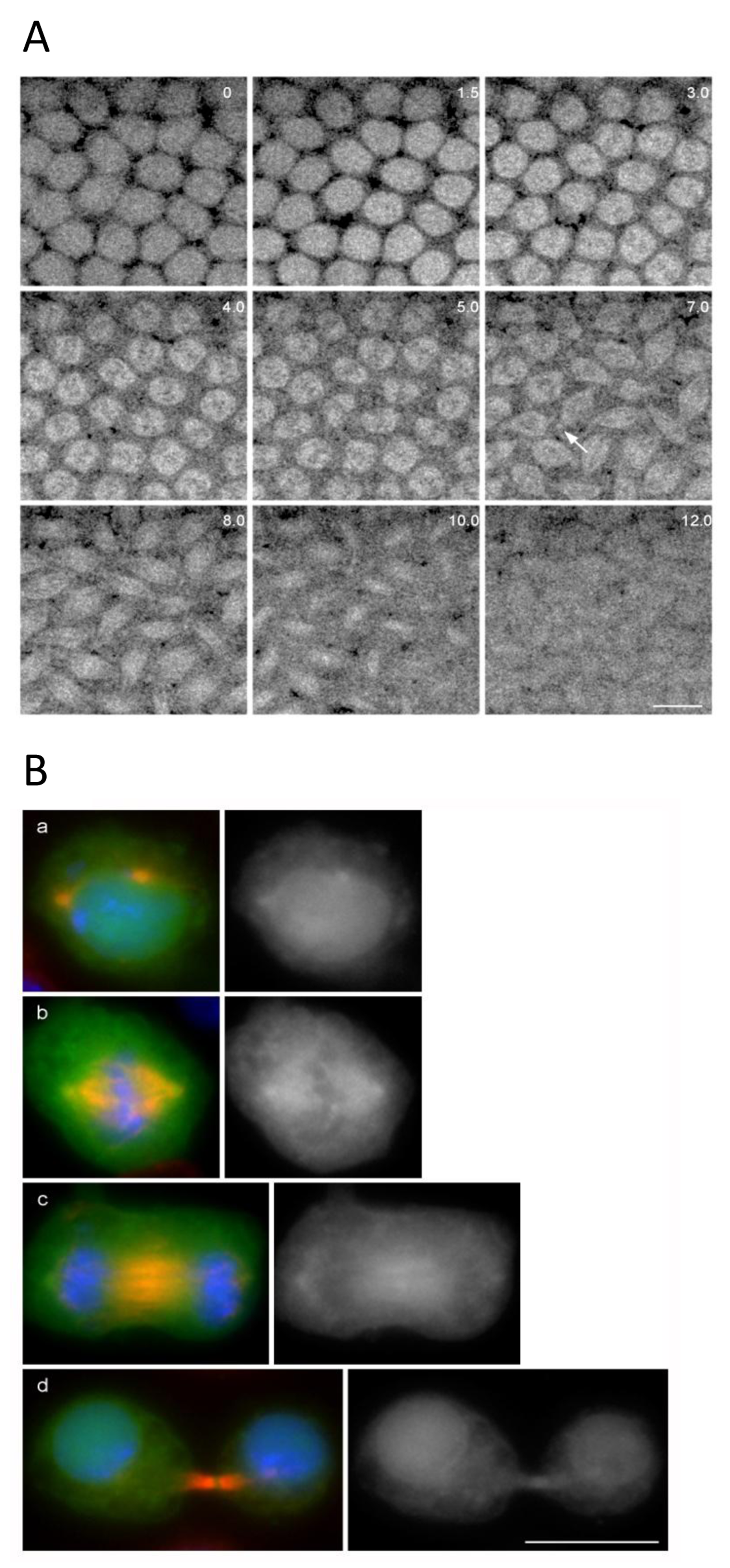

Supplement: Figure S3 — Localisation of Endos in syncytial embryos and cultured DMEL cells. A. In flies transgenic for endos-EGFP, green fluorescence is seen mainly within the area bounded by the spindle envelope and also accumulates around centrosomes (arrow in panel 7.0 minutes). Endos-EGFP fluorescence intensity increases prior to the formation of the spindle (compare panels 0, 1.5 and 3.0 minutes), remains high throughout metaphase (panels 4.0 to 7.0 minutes), but appears to decrease after anaphase (compare panel 8.0 and 10.0 minutes) and remains low until the next mitotic division. Note the low levels of cytoplasmic Endos-EGFP throughout division. Time is indicated in minutes. Scale bar represents 10 µm. B. In DMEL cells expressing endos-EGFP, fluorescence is present both in the nucleus and around the centrosomes in prophase cells (panel a). It localises to the spindle region at metaphase (panel b) and its level decreases following anaphase (panel c; note that it is still associated with the central spindle). Endos-EGFP is also present at lower levels in the cytoplasm throughout mitosis. Cells are stained to reveal microtubules in red, Endos-GFP in green, and DNA in blue. Scale bar represents 10 µm. (TIF) [file pgen.1002225.s003.tif]

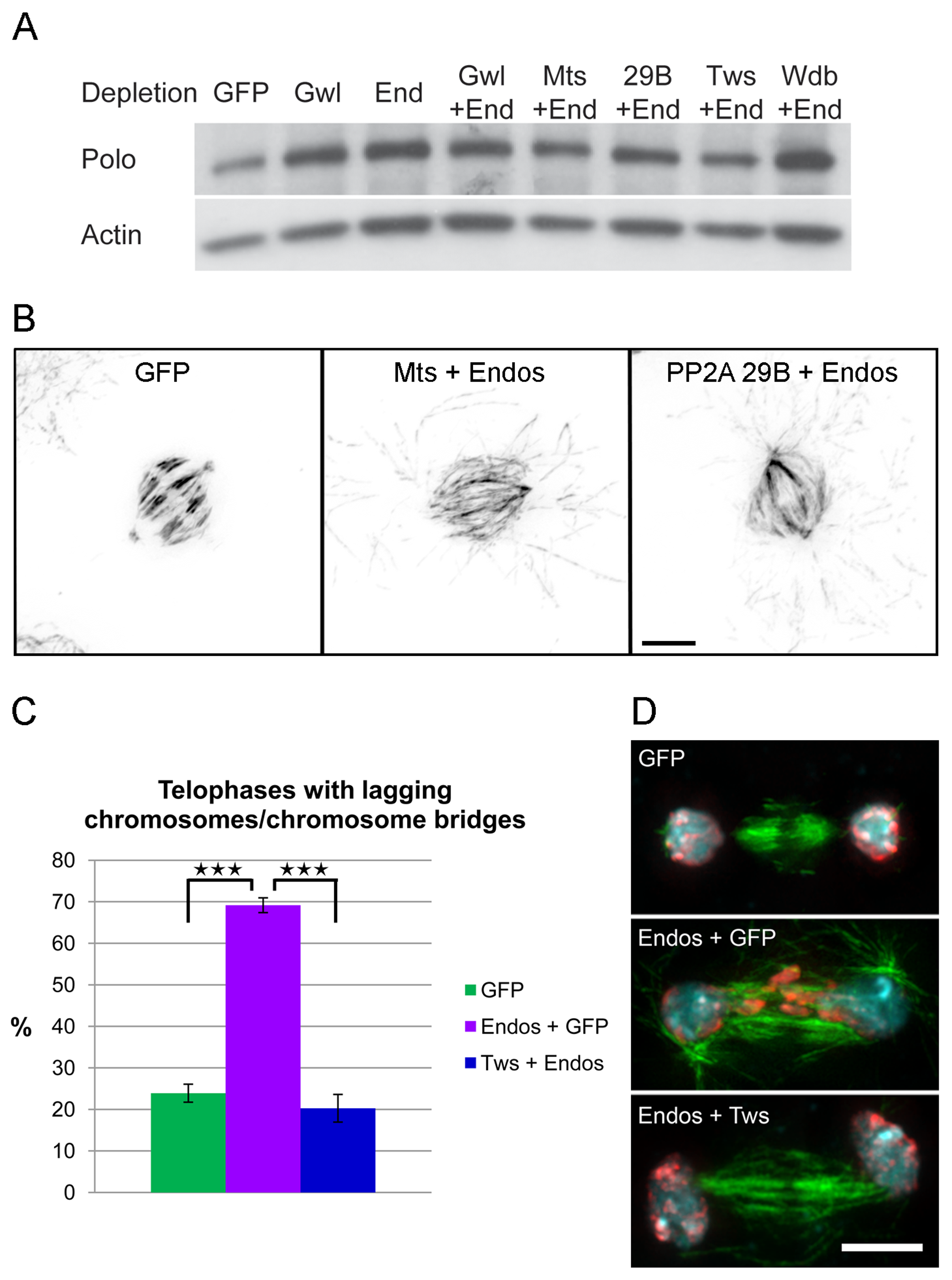

Supplement: Figure S4 — Phenotypes of DMEL cells after Endos and PP2A combined depletion. A. Depletion of Endos, alone or in combination with PP2A subunits, minimally affects levels of Polo kinase at best. Levels of proteins are visualised by Western blot after single or double depletion of DMEL cells for GFP (control depletion), Greatwall (Gwl), Endos (End) and the various subunits of PP2A: Microtubule Star (Mts), 29B, Twins (Tws) or Widerborst (Wdb). Extracts were analysed with antibodies detecting Polo and Actin (loading control). B. Additional phenotypes after depletion of Endos and PP2A subunits. After double depletion of Endos and either the catalytic subunit (Mts) or the structural subunit (PP2A 29B) of PP2A, (pro)metaphases show some long microtubules that are not captured by the spindle and may or may not be astral microtubules. Scale bar represents 5 µm. C. Lagging chromosomes and chromosome bridges after endos depletion are suppressed by PP2A-twins/B55 knockdown. Mitotic defects following Endos RNAi treatment were scored as proportions of telophases with lagging chromosomes and/or chromosome bridges. The endos knockdown phenotype is suppressed when the PP2A-twins subunit is also knocked down. Error bars represent sem of three independent experiments. P values are from a Student's T-test with *** = p<0.001; a minimum of 200 telophases were scored per treatment. D. Representative mitotic figures for the above depletions as indicated. Scale bar represents 5 µm. (TIF) [file pgen.1002225.s004.tif]

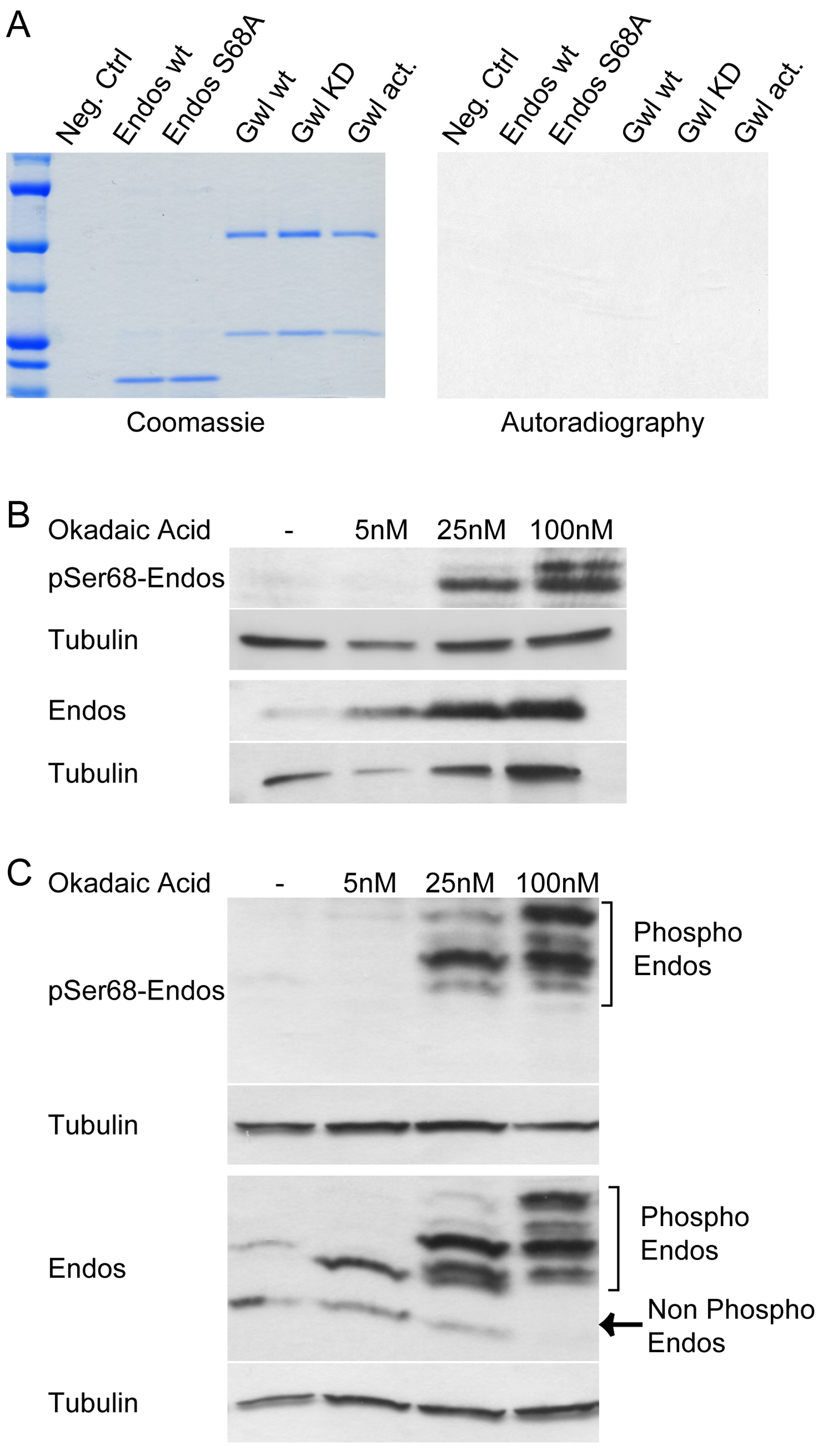

Supplement: Figure S5 — Phosphorylation of Endos. A. Proteins used in the phosphorylation assay. Kinases immunoprecipitated from cell extracts (Myc-Greatwall wild type (Gwl wt), Myc-Greatwall kinase dead (Gwl KD, mutated for Lys87Arg) or Myc-Greatwall hyperactive form (Gwl act., mutated for Lys97Met)) and Endos substrates (wild type (Endos wt) or mutated for Ser68Ala (Endos S68A)) were resolved independently on SDS-PAGE. The gel was stained with Coomassie Blue (left panel), and the 32P-labeled proteins were visualised by autoradiography (right panel). None of the proteins used in the phosphorylation assay become labelled by 32P as detected by autoradiography indicating the absence of non-specific phosphorylation. Levels of Greatwall immunoprecipitated were also analysed by Western blot (data not shown and published in [4]). B. Endos is phosphorylated at Ser68 in cells and phosphorylation is enhanced by treatment with okadaic acid. DMEL cells were treated with none or the indicated concentrations of okadaic acid for 2 hours before preparation of cells extracts. The extracts were analysed on independent Western blots using antibodies detecting either Endos phosphorylated at Ser68 (upper panels) or Endos (lower panels) and tubulin (loading control). Phosphorylation of Endos at Ser68 is observed in cells and is increased after treatment with 25 nM okadaic acid (B upper panel). Such okadaic acid treatment also leads to an increase of the total Endos level in cells (B lower panel). C. Endos is phosphorylated at Ser68 in addition to other amino acid residues in cells. The same extracts as described in B were analysed on SDS-PAGE gels in the presence of Phos-tag instead of regular SDS-PAGE gels. The shift in Endos mobility that is seen after treatment with 5 nM okadaic acid suggests a block to dephosphorylation by PP2A. This particular modification is not recognised by the anti-P-Ser68 antibody. This antibody does, however, recognise a form of Endos that appears after treatment with 25 nM okadaic [file pgen.1002225.s005.tif]
