# Supplementary material for: Suppression of Scant Identifies Endos as a Substrate of Greatwall Kinase and a Negative Regulator of Protein Phosphatase 2A in Mitosis
Source: PLoS Genet. 2011 Aug 11;7(8):e1002225. doi: 10.1371/journal.pgen.1002225 (PMC3154957; doi:10.1371/journal.pgen.1002225)
Supplement: Table S1 — Mitotic defects in endos mutants. Mitosis in cells of fixed preparations of larval central nervous systems of the indicated genotype were analysed according to the following criteria: mitotic index (MI), Metaphase∶Anaphase (M∶A) ratio and percentage of Anaphase defects. The deficiency indicated Df corresponds to the deficiency Df(3L)fz-GF3b. Extreme chromosome bridging made it difficult to recognise anaphase cells in endos67006/Df(3L)fz-GF3b and CG6650/CyO; endos67006/Df(3L)fz-GF3b and could account for the very high assessment of the Metaphase∶Anaphase ratio in these lines. (DOC) [file pgen.1002225.s006.doc]

**Table S1. Mitotic defects in *endos* mutants.**

| Allele | # brains | # cells | MI (%) | M:A ratio | % Anaphase defects |
| --- | --- | --- | --- | --- | --- |
| Oregon R | 4 | 8076 | 1.4 | 4:1 | 0.0% |
| *endos^+^/CyO; endos^67006^/Df* | 1 | 2024 | 1.4 | 4:1 | 0.0% |
| *CG6650/CyO ; endos^67006^/Df* | 4 | 8085 | 2.0 | 19:1 | 25.0% |
| *endos^67006^/Df* | 4 | 8210 | 1.9 | 21:1 | 29.0% |
| *endos^79^/Df* | 4 | 8243 | 2.0 | 6:1 | 8.7% |
| *endos^60^/Df* | 4 | 8164 | 2.5 | 10:1 | 33.3% |
| *endos^1^/Df* | 3 | 6111 | 2.4 | 11:1 | 50.0% |
