# Supplementary material for: Suppression of Scant Identifies Endos as a Substrate of Greatwall Kinase and a Negative Regulator of Protein Phosphatase 2A in Mitosis
Source: PLoS Genet. 2011 Aug 11;7(8):e1002225. doi: 10.1371/journal.pgen.1002225 (PMC3154957; doi:10.1371/journal.pgen.1002225)
Supplement: Table S2 — Quantitation of mitotic defects following endos RNAi. Cultured cells depleted for Endos or GFP (control) were analysed for several mitotic parameters: mitotic index (MI), percentage of spindle defects, percentage of chromosome defects and the relative percentage of Telophase/Cytokinesis cells. A total of 281,363 GFP-depleted cells were scored of which 9,506 were in mitosis. A total of 240,442 Endos depleted cells were scored of which 18,688 were in mitosis. (DOC) [file pgen.1002225.s007.doc]

**Table S2. Quantitation of mitotic defects following *endos* RNAi.**

|  | MI (%) | Spindle defects | Chromosome defects | Telophase/Cytokinesis |
| --- | --- | --- | --- | --- |
| *GFP* RNAi | 1.0 | 8% | 12% | 39% |
| *endos* RNAi | 2.4 | 25% | 84% | 15% |
