# Supplementary material for: Suppression of Scant Identifies Endos as a Substrate of Greatwall Kinase and a Negative Regulator of Protein Phosphatase 2A in Mitosis
Source: PLoS Genet. 2011 Aug 11;7(8):e1002225. doi: 10.1371/journal.pgen.1002225 (PMC3154957; doi:10.1371/journal.pgen.1002225)
Supplement: Table S3 — Details of DMEL cells expressing GFP-Cid and β-tubulin-mRFP analysed by time lapse imaging. Cells expressing GFP-Cid and β-tubulin-mRFP were treated with endos dsRNA or untreated and analysed by time lapse imaging. The table reports the duration of the prometaphase for each cell recorded. The table also indicates whether nuclear envelope breakdown and anaphase could be observed during the duration of the recording. (DOC) [file pgen.1002225.s008.doc]

**Table S3. Details of DMEL cells expressing GFP-Cid and β-tubulin-mRFP analysed by time lapse imaging.**

| Control | | | | Endos depletion | | | |
| --- | --- | --- | --- | --- | --- | --- | --- |
| cell | Prometaphase (min) | NEBD | anaphase | cell | Prometaphase (min) | NEBD | anaphase |
| 1 | 54 | yes | yes | 1 | >155 | n.o.* | n.o. |
| 2 | 40 | yes | yes | 2 | >50 | n.o. | yes |
| 3 | 38 | yes | yes | 3 | >170 | n.o. | n.o. |
| 4 | 32 | yes | yes | 4 | 75 | yes | yes |
| 5 | 46 | yes | yes | 5 | >190 | yes | n.o. |
| 6 | 26 | yes | yes | 6 | >50 | n.o. | yes |
| 7 | 26 | yes | yes | 7 | >155 | yes | n.o. |
| 8 | 50 | yes | yes | 8 | 130 | yes | yes |
| 9 | 28 | yes | yes | 9 | 140 | yes | yes |
| 10 | 32 | yes | yes |  |  |  |  |
| 11 | 58 | yes | yes |  |  |  |  |

* n.o.: not observed. NEBD: nuclear envelope breakdown.
