# Supplementary material for: Suppression of Scant Identifies Endos as a Substrate of Greatwall Kinase and a Negative Regulator of Protein Phosphatase 2A in Mitosis
Source: PLoS Genet. 2011 Aug 11;7(8):e1002225. doi: 10.1371/journal.pgen.1002225 (PMC3154957; doi:10.1371/journal.pgen.1002225)
Supplement: Table S4 — Primers list. Summary of the primers used during this work. Note that dsRNA primers directed against subunits of PP2A are identical to the ones published in [33]. (DOC) [file pgen.1002225.s009.doc]

**Table S4**. **Primers list.**

| **Gateway primers** | |
| --- | --- |
| Endos for N-ter fusion entry clone: HR184_Endos_FL_for | GGGGACAAGTTTGTACAAAAAAGCAGGCTTCACCATGAGCTCCGCGGAAGAAAACAGC |
| Endos for N-ter fusion entry clone: HR186_Endos_FL_Stop_rev | GGGGACCACTTTGTACAAGAAAGCTGGGTCTTAGCTCGTCGCCGGGAACTTGT |
| Endos for N-ter fusion entry clone with thrombin site: HR188_Endos_FLThrombNter_for | GGGGACAAGTTTGTACAAAAAAGCAGGCTTCctggttccgcgtggatccAGCTCCGCGGAAGAAAACAGC |
| **dsRNA Primers** | |
| dsRNA Greatwall: HR160_Gwl_T7_265ex2 _for | TAATACGACTCACTATAGGGAGAATGCGCAAGTCAGAGATG |
| dsRNA Greatwall: HR161_Gwl_T7_687ex2_rev | TAATACGACTCACTATAGGGAGAGTAAGCGACAAAAGCTGG |
| dsRNA ORF Endos: HR164_Endos_T7_minus10ex1_for | TAATACGACTCACTATAGGGAGAGCAGCACACAATGAGCTC |
| dsRNA ORF Endos: HR165_Endos_T7_338ex2_rev | TAATACGACTCACTATAGGGAGAGTCGCCGGGAACTTGT |
| dsRNA 3’UTR Endos: En3UF | TAATACGACTCACTATAGGGAGACACCACTCAACACTCACCTC |
| dsRNA 3’UTR Endos: En3UR | TAATACGACTCACTATAGGGAGACCAGTAATGTACGAGAACGTG |
| dsRNA PP2A-Mts: HR170_PP2Amts_T7_3UTR_for | TAATACGACTCACTATAGGGAG CCTACGCAGCTTACATTTACACATA |
| dsRNA PP2A-Mts: HR171_ PP2Amts_T7_3UTR_rev | TAATACGACTCACTATAGGGAGATAGGTTCGATTGGATTGTATCATTT |
| dsRNA PP2A-29B: HR174_PP2A29B_T7_254Ex3_for | TAATACGACTCACTATAGGGAG CAGAGTTTGCCATGTACTTGATTC |
| dsRNA PP2A-29B: HR175_ PP2A29B_T7_626ex3_rev | TAATACGACTCACTATAGGGAGAGGAATCAAATCGGACTTCAGATACT |
| dsRNA PP2A-Twins:HR178_PP2Atws_T7_Ex3_for | TAATACGACTCACTATAGGGAGACTGATCCGGGATCCACAGAATGTAA |
| dsRNA PP2A-Twins: HR179_ PP2Atws_T7_Ex3_rev | TAATACGACTCACTATAGGGAGACACACTTTGATGCTCAAGTAATCCC |
| dsRNA PP2A-Widerborst: HR180_PP2Awdb_T7_980Ex5_for | TAATACGACTCACTATAGGGAGATCGATCCGCCGCAGTTTGTCAAGAT |
| dsRNA PP2A-Widerborst: HR181_ PP2Awdb_T7_1004Ex7_rev | TAATACGACTCACTATAGGGAGATCGACTGCTGCTGATGAGAGTTCAG |
| dsRNA PP2A-B’: HR217_PP2AB’_T7_2ex8_for | TAATACGACTCACTATAGGGAGAAGCCTACTTAAATTTTGGCCC |
| dsRNA PP2A-B’: HR218_PP2AB’_T7_393ex8_rev | TAATACGACTCACTATAGGGAGAATCTGCTTCTCTTGCTTGTAGTTC |
| dsRNA PP2A-B’’: HR221_PP2AB’’_T7_156ex1_for | TAATACGACTCACTATAGGGAGATTCCAAGGTGTCCAAAGCGCAGGGA |
| dsRNA PP2A-B’’: HR222_ PP2AB’’_T7_657ex1_rev | TAATACGACTCACTATAGGGAGACAGATTCTGGGTTTGCTGCGAGGAG |
| dsRNA GFP Control: GFPT7F | TAATACGACTCACTATAGGGAGACTTCAGCCGCTACCCC |
| dsRNA GFP Control: GFPT7R | TAATACGACTCACTATAGGGAGATGTCGGGCAGCACG |
| **Directed mutagnesis primers** | |
| HR154_Endos_S68A_for | GCAAAAGTTCTTCGACGCGGGCGATTACCAG |
| HR155_Endos_S68A_rev | CTGGTAATCGCCCGCGTCGAAGAACTTTTGC |
| HR156_Endos_S68D_for | GGGCAAAAGTTCTTCGACGACGGCGATTACCAGATGGC |
| HR157_Endos_S68D_rev | GCCATCTGGTAATCGCCGTCGTCGAAGAACTTTTGCCC |
| **Cloning primers** | |
| CG6513 rescue construct: CG6513gen-for2 | TAGCGGCCGCTATGTTGCTTCCGTTGGTAGAGG |
| CG6513 rescue construct: CG6513gen-rev2 | ATGCGGCCGCTGGCGTTTATCTGCAAGTCC |
| CG6650 rescue construct: CG6650gen-for | TAGCGGCCGCTTCTCGAGATCCGTGAGGTTAG |
| CG6513 rescue construct: CG6650gen-rev | TAGCGGCCGCGTTGGTCCTCATCCTGTTCCG |
| pCasPer4-Endos-EGFP : EGFP for | ATTGATCAGCATGGTGAGCAAGGGCGAGGAG |
| pCasPer4-Endos-EGFP : EGFP rev | TCTGATCACCTTGTACAGCTCGTCCATGCCGAG |
